# Ulipristal acetate versus levonorgestrel-releasing intrauterine system for heavy menstrual bleeding (UCON): a randomised controlled phase III trial

**DOI:** 10.1016/j.eclinm.2023.101995

**Published:** 2023-05-18

**Authors:** Lucy H.R. Whitaker, Lee J. Middleton, Jane P. Daniels, Alistair R.W. Williams, Lee Priest, Smita Odedra, Versha Cheed, Clive E. Stubbs, T. Justin Clark, Mary-Ann Lumsden, Dharani K. Hapangama, Siladitya Bhattacharya, Paul P. Smith, Elaine P. Nicholls, Neil Roberts, Scott I. Semple, Lucky Saraswat, Jane Walker, Rohan R. Chodankar, Hilary O.D. Critchley, UCON Collaborative

**Affiliations:** a MRC Centre for Reproductive Health, University of Edinburgh, Edinburgh, UK; b Birmingham Clinical Trials Unit, University of Birmingham, Birmingham, UK; c Nottingham Clinical Trials Unit, University of Nottingham, Nottingham, UK; d Division of Pathology, University of Edinburgh, Edinburgh, UK; e Birmingham Women's and Children's Hospital, Birmingham, UK; f Reproductive & Maternal Medicine, University of Glasgow, Glasgow, UK; g Department of Women's and Children's Health, University of Liverpool, Liverpool, UK; h University of Aberdeen, Aberdeen, UK; i Adcal H.R. Consultancy, UK (PPI representative); j BHF Centre for Cardiovascular Science, University of Edinburgh, Edinburgh, UK; k Department of Clinical Radiology, Royal Infirmary of Edinburgh, Edinburgh, UK

**Keywords:** Heavy menstrual bleeding, Ulipristal acetate, Selective progesterone receptor modulator, Levonorgestrel-releasing intrauterine system, Randomised controlled trial, Fibroid, Leiomyoma, Adenomyosis endometrium, Uterus, Quality of life, Amenorrhoea, Ultrasound, Progesterone receptor modulator associated endometrial changes, Drug induced liver injury, Urgent safety measures

## Abstract

**Background:**

Heavy menstrual bleeding affects one in four women and negatively impacts quality of life. Ulipristal acetate is prescribed to treat symptoms associated with uterine fibroids. We compared the effectiveness of ulipristal acetate and the levonorgestrel-releasing intrauterine system at reducing the burden of heavy menstrual bleeding, irrespective of the presence of fibroids.

**Methods:**

This randomised, open-label, parallel group phase III trial enrolled women over 18 years with heavy menstrual bleeding from 10 UK hospitals. Participants were centrally randomised, in a 1:1 ratio, to either three, 12-week treatment cycles of 5 mg ulipristal acetate daily, separated by 4-week treatment-free intervals, or a levonorgestrel-releasing intrauterine system. The primary outcome, analysed by intention-to-treat, was quality of life measured by the Menorrhagia Multi-Attribute Scale at 12 months. Secondary outcomes included menstrual bleeding and liver function. The trial is registered with ISRCTN, 20426843.

**Findings:**

Between June 5th, 2015 and February 26th, 2020, 236 women were randomised, either side of a recruitment suspension due to concerns of ulipristal acetate hepatoxicity. Subsequent withdrawal of ulipristal acetate led to early cessation of recruitment but the trial continued in follow-up. The primary outcome substantially improved in both groups, and was 89, (interquartile range [IQR] 65 to 100, n = 53) and 94, (IQR 70 to 100, n = 50; adjusted odds ratio 0.55, 95% confidence interval [CI] 0.26–1.17; p = 0.12) in the ulipristal and levonorgestrel-releasing intrauterine system groups. Rates of amenorrhoea at 12 months were higher in those allocated ulipristal acetate compared to levonorgestrel-releasing intrauterine system (64% versus 25%, adjusted odds ratio 7.12, 95% CI 2.29–22.2). Other outcomes were similar between the two groups and there were no cases of endometrial malignancy or hepatotoxicity due to ulipristal acetate use.

**Interpretation:**

Our findings suggested that both treatments improved quality of life. Ulipristal was more effective at inducing amenorrhoea. Ulipristal has been demonstrated to be an effective medical therapeutic option but currently its use has restrictions and requires liver function monitoring.

**Funding:**

10.13039/501100000265UK Medical Research Council and National Institute of Health Research EME Programme (12/206/52).

**Research in context**

**Evidence before this study**

The ECLIPSE trial showed that the levonorgestrel-releasing intrauterine system when compared to usual medical therapies was the most cost-effective and gave the greatest improvement in women's assessment of the effect on HMB. Selective progesterone receptor modulators, including ulipristal acetate have shown efficacy in reducing uterine fibroid size and control of bleeding compared to a placebo but there had been no direct comparison between levonorgestrel-releasing intrauterine systems and selective progesterone receptor modulators in women with or without fibroids before this trial. During this study an update of the data from the 2017 Cochrane Review “Selective progesterone receptor modulators (SPRMs) for uterine fibroids”, was conducted by a systematic search of Pubmed and trial and review registers, using the terms “ulipristal” and “fibroid” (Oct 1, 2022), which did not identify any additional published direct or network comparisons of selective progesterone receptor modulators.

**Added value of this study**

This is the only randomised trial of ulipristal acetate compared to another medical treatment for the symptom of heavy menstrual bleeding in women with no or small fibroids. The trial did not find evidence of a difference in quality of life but ulipristal acetate was superior to the levonorgestrel-releasing intrauterine system at inducing amenorrhoea. Two drug safety alerts first altered, then resulted in the premature termination of recruitment, and had the potential to introduce response bias, meaning the trial did not reach its target sample size.

**Implications of all the available evidence**

This study suggests that ulipristal acetate treatment achieves amenorrhoea, improves quality of life and has a high rate of patient satisfaction in women with HMB, with no or small fibroids, reflecting those observed in women with significant fibroids. Due to concerns about hepatotoxicity, the use of ulipristal acetate is limited to those women who cannot have surgery or uterine artery embolisation, or with refractory bleeding where other procedures have failed, and then only with appropriate counselling and monitoring for signs and symptoms of liver damage. The development of new selective progesterone receptor modulators is needed to address the unmet need for an acceptable and safe oral treatment for heavy menstrual bleeding.

## Introduction

Heavy menstrual bleeding (HMB), defined as excessive menstrual blood loss which interferes with a woman's physical, social, emotional, and/or material quality of life,^1^ is reported to affect 1 in 4 women of reproductive age,^2^ and extent and burden of symptoms may be under-reported.^3^ The underlying causes and mechanisms of HMB are multifactoria^4^ and broadly classified into structural (including uterine fibroids) and non-structural causes.^5^ Management of HMB is often driven by factors including age, desire for fertility preservation, clinician and patient preference, rather than aetiology.^6^ The levonorgestrel-releasing intrauterine system is recommended as the first-line treatment for HMB in clinical guidelines^1^ as it substantially reduces menstrual blood loss, often resulting in amenorrhoea. However, unscheduled bleeding may be problematic,^7^ it is a contraceptive, fitting of the system can be unacceptable^8^ and the presence of fibroids may increase expulsion rates.^9^

Selective Progesterone Receptor Modulators (SPRMs) may provide a medical solution as progesterone plays a pivotal role in both menstruation and fibroid growth. In women with uterine fibroids ranging from 3 to 10 cm in size treated with ulipristal acetate the control of HMB was achieved in over 90% of women and amenorrhoea reported in 70%, though mechanisms through which the bleeding control is achieved remain poorly understood.^10^^,^^11^ Ulipristal was well tolerated and reported side-effects were limited to minor complaints such as headache and breast tenderness.^10^^,^^11^ Though high dose (30 mg) ulipristal acetate is licensed as an emergency contraceptive, the dosage of ulipristal acetate licensed for treatment of heavy menstrual bleeding (5 mg daily) is not licensed as a contraceptive.^12^ Consequently, in contrast to the levonorgestrel-releasing intrauterine system, which confers contraception in addition to management of HMB, those using the ulipristal acetate dosage licensed for treatment of heavy menstrual bleeding are recommend to use a non-hormonal contraceptive method during treatment.^12^ Despite therapeutic potential, robust data on the long-term effectiveness and mechanisms of action of SPRMs in women with HMB and either small, or no fibroids, remain unknown.

The aim of the Ulipristal acetate versus Conventional treatment (UCON) trial was to evaluate the safety, tolerability and effectiveness of ulipristal acetate on HMB symptoms in those with small or no fibroids, in comparison to a levonorgestrel-releasing intrauterine system.

## Methods

The UCON trial was a randomised, open-label, parallel group, multi-centre phase III trial of ulipristal acetate compared with levonorgestrel-releasing intrauterine system in women presenting to primary and/or secondary care with HMB. The study team recognises that people have diverse gender identities, and in this study report, the word ‘woman’ is used to describe patients or individuals whose sex assigned at birth was female, whether they identify as female, male, or non-binary and who reported heavy menstrual bleeding. Ten UK hospitals participated. The trial had a favourable ethical opinion from the London (Bloomsbury) National Research Ethics Service Committee (REC No 14/LO/1602) and clinical trial authorisation from the MHRA. A Trial Steering Committee (TSC) provided independent oversight and a Data Monitoring Committee (DMC) reviewed confidential data throughout the course of the trial. Data was verified by the trial statisticians. All versions of the protocol can be accessed here https://fundingawards.nihr.ac.uk/award/12/206/52. The trial was registered with ISRCTN, 20426843.

The UCON trial opened to recruitment on 31st March 2015. In February 2018 and March 2020, the trial was subject to two urgent safety measures as a consequence of drug alerts issued by the European Medicines Agency (EMA) and UK Medicines and Healthcare products Regulatory Agency (MHRA) following reports of serious liver injury in patients receiving ulipristal acetate treatment. Amendments to the protocol, consequent upon the two urgent safety measures, and based on the MHRA guidance to monitor the safety of existing and new participants, were enacted.

Recruitment of participants to UCON was suspended on 12th February 2018 and restarted on the 18th October 2018. Follow-up assessments and time-points were modified as a result and became effective from 20th March 2018. In March 2020, the EMA temporarily suspended use of ulipristal acetate for a second time, whilst a further safety review was undertaken. Trial recruitment was again suspended. This second urgent safety measure coincided with the COVID-19 pandemic, which resulted in the suspension of non-urgent public health related clinical research in the UK. At this time, many routine gynaecology clinical services were halted and whilst follow-up and telephone monitoring of existing participants continued, participants were not required to attend hospital for trial clinical procedures, unless there was clinical concern. When the marketing authorisation for ulipristal acetate was revoked by the EMA in September 2020, it was inevitable that the trial would not reopen to recruitment.

All existing participants completed any missed clinical procedures and the final follow-up of participants completed on 31st May 2021.

### Participants

Participants were recruited from gynaecology outpatient departments. At a screening visit, a transvaginal and/or abdominal ultrasound scan was conducted (unless an adequate ultrasound scan report within the three months prior to randomisation was available) and an endometrial biopsy taken (unless an adequate endometrial biopsy had been taken within the previous six months). Clinical history was elicited and a menstrual blood loss diary was provided to the participant. Eligibility was confirmed at a subsequent appointment at least one menstrual cycle after the screening visit.

Eligibility criteria were women aged ≥18 years, with menstrual bleeding perceived by the patient to be heavy and troublesome, willing to use barrier contraception if allocated to ulipristal acetate and who gave written informed consent to trial participation and procedures. Exclusion criteria were, a >14-week fibroid uterus and/or cavity length >11 cm or submucosal fibroids >2 cm diameter seen on an ultrasound scan, contraindications to, or special warnings for, administration of ulipristal acetate or fitting of a levonorgestrel-releasing intrauterine system, intention to use or continue current use of standard medical treatments for HMB; a past, current or suspected diagnosis of endometrial hyperplasia or uterine, cervical, ovarian or breast cancer, pregnant, breastfeeding or planning to become pregnant within 12 months. From 20th March 2018, hepatic impairment, defined as levels of alanine transaminase or aspartate aminotransferase of more than two times the upper limit of normal precluded participation.

### Randomisation and masking

Participants were randomised using a secure online randomisation service in an equal ratio to ulipristal acetate or levonorgestrel-releasing intrauterine system using the following minimisation variables: age (≤35yrs or >35yr), body mass index (≤25 kg/m2 or >25 kg/m^2^), presence of any fibroid >2 cm, duration of symptoms (<1 year or ≥1 year) and recruitment sites. As the treatments differed in route of administration, the participants, investigators, research nurses and other attending clinicians were not blinded to the treatment allocation.

### Procedures

A levonorgestrel-releasing intrauterine system, either Mirena™ (Bayer Plc) or Levosert (Actavis UK), with a daily dose equivalent to 20 μg levonorgestrel was fitted according to manufacturers’ recommendations. Participants were counselled to expect some disturbance to menstrual bleeding patterns and advised the levonorgestrel-releasing intrauterine system could remain in-situ up for up to five years. The levonorgestrel--releasing intrauterine system was chosen as comparator as this is the first line treatment recommended by the UK NICE guidelines for the management of heavy menstrual bleeding.^1^

Participants allocated ulipristal acetate (Esmya™, Gedeon Richter) were instructed to take 5 mg tablets daily in three courses of 12 weeks each, with four weeks off-treatment interval when a light vaginal withdrawal bleed may occur. After 20th March 2018, participants taking ulipristal acetate provided monthly blood samples, including 2–4 weeks after each ulipristal acetate course, for liver function tests including alanine transaminase and aspartate aminotransferase, and other tests used in local protocols. Participants were required to cease taking ulipristal acetate, or not restart a new cycle, if either alanine transaminase or aspartate aminotransferase were greater than three times the upper limit of normal.^13^ After the second urgent safety measure, when all participants were advised to stop ulipristal acetate treatment, some liver function tests were delayed, although contact was made by telephone to ask about symptoms of liver injury. Throughout, anyone with signs or symptoms suggestive of liver injury stopped their ulipristal acetate treatment, were closely monitored and referred for specialist hepatology evaluation if clinically indicated.

Following the first urgent safety measure, those participants prescribed ulipristal acetate were allowed to complete their current course of treatment, but not start any subsequent course. The second USM required participants taking ulipristal acetate to cease treatment immediately and not take any further courses. The trial continued with follow-up of participants, regardless of adherence (enforced or non-enforced) to 12-months post randomisation.

### Outcomes

The primary outcome was the Menorrhagia Multi-Attribute Scale (MMAS) questionnaire score,^14^ designed and validated to capture the impact of HMB on women's day-to-day life, at 12 months post-randomisation. Summary scores range from 0 (worst affected) to 100 (not affected).

Participant reported secondary outcomes were: menstrual cycle regularity (ordinal four-point scale); duration of period (ordinal three option scale); menstrual bleeding, captured and categorised by validated Pictorial Blood Loss Assessment Chart, (PBAC),^15^ pelvic pain during periods, intercourse and at other times, using a visual analogue scales, the Uterine Fibroid Symptom and Quality of Life (UFS-QoL) instrument (only for individuals diagnosed with fibroids^16^), the Sexual Activity Questionnaire (SAQ)^17^ and generic quality of life (EQ-5D-5L).^18^ Due to the differing treatment schedules, the MMAS, UFS-QoL, SAQ and EQ-5D-5L were completed in the final week of each ulipristal acetate cycle, whilst the PBAC was completed over the final four weeks of each treatment cycle and the first four weeks off treatment. Those in the levonorgestrel-releasing intrauterine system group completed the questionnaires at three, six and 12 months after randomisation. Other secondary outcomes, completed at 12 months included: satisfaction with treatment outcome measured on a five-point Likert scale; participant rating of effect of treatment on HMB over 12 months measured on a four-point Likert scale; whether participant was willing to recommend the treatment to a friend; surgical intervention (hysterectomy, endometrial ablation and other gynaecological surgery).

Other clinical measurements derived from pelvic ultrasound, haemoglobin and serum oestradiol will be reported elsewhere.

An endometrial biopsy was undertaken prior to randomisation and at four weeks (or as soon as feasible during the Covid-19 restrictions) after cessation of ulipristal acetate treatment. Participants in the levonorgestrel-releasing intrauterine system group did not have a biopsy. A diagnosis of normal, benign, hyperplasic or malignant) and further categorisation of PRM-associated or other non-physiological changes in the ulipristal acetate group only was made. If PRM-associated changes were observed a repeat endometrial biopsy was performed at 13 weeks post treatment cessation, and repeated as close to 26 weeks, as possible, if these changes persisted.

Participants in the ulipristal acetate group provided their adherence using predefined categories. They were considered adherent if ulipristal acetate was taken every day or most days. Pill counting was considered unfeasible due to the duration of treatment. Reasons for change or cessation of treatment were collected, including decisions driven by perceived side effects of treatment such as weight gain. Retention of the levonorgestrel-releasing intrauterine system was captured by self-report; participants were considered adherent provided they did not report removal of the system. Postal questionnaires collected information on admission to hospital, gynaecological investigations or treatments, relevant diagnoses, or use of any new medications. Pregnancy was considered an adverse event if the participant was compliant with either trial treatment, but not if they intentionally stopped treatment.

A serious adverse event was defined as any event or reaction that was life-threatening, required emergency hospitalisation, resulted in death or persistent or significant disability or if a congenital anomaly was diagnosed in the foetus of a participant who became pregnant. All diagnoses of endometrial cancer, ovarian cancer, cervical cancer, breast cancer or ductal carcinoma were defined as serious adverse events. The local investigator had to assign seriousness, severity, causality and expectedness (if deemed related) to the serious adverse event before reporting. Those categorised by the local investigator as both suspected to be related to the trial drugs and unexpected, were subject to expedited reporting.

### Statistical analysis

The enforced non-compliance because of the temporary (and subsequently permanent) withdrawal of ulipristal acetate had substantial implications for the sample size and validity of the data reported by participants. Therefore it was necessary to redefine the analysis populations, considering the urgent safety measures might influence their responses, as well as any other new biases that may arise in either group due to safety concerns around ulipristal acetate. Any changes to the Statistical Analysis Plan arising from the impact of the urgent safety measures were approved by a statistician independent to the trial to ensure they were not driven by knowledge of the accruing data.

The original planned primary analysis population comprised all participants, regardless of adherence to treatment, in keeping with the principles of intention-to-treat. The revised primary analysis population (population A, Fig. S1) comprised participants with questionnaire responses received prior to 12th February 2018, along with questionnaire responses from participants recruited in the second phase following the study restart on 18th October 2018, provided these were returned before the 18th March 2020. There was considered to be no additional risk of bias in respect of these participants as a result of the urgent safety measures, although whether those randomised in the second phase were completely comparable with the earlier population, given they were informed of the risk of liver damage by ulipristal acetate was a residual concern. We planned to investigate any potential impact of this through examination of interaction of treatment effect by recruitment period. The same approach would be used for all assessment time-points. Participant responses were to be included regardless of adherence to treatment in keeping with principles of intention-to-treat, to limit any potential for confounding biases. The supportive analysis populations are described further in Fig. S1.

An evaluation of the effectiveness of levonorgestrel-releasing intrauterine system against standard treatment for HMB, using MMAS as the primary outcome, demonstrated a difference of 13 points between the groups with a standard deviation of 24 points.^7^ This difference was considered to be clinically meaningful^19^ and is equivalent to approximately 0.5 standard deviations. To detect a difference of this size with 90% power (p = 0.05) required 86 participants in each group (172 in total). To allow for a 20% loss to follow-up or pregnancy, the sample size was inflated to 220 participants.

Prior to the first halt of recruitment, the trial had recruited 198 participants, of whom 89 had provided a primary outcome response at 12 months. Upon restart, the aim then was to obtain 172 primary outcomes responses at 12 months in the primary analysis population, requiring inflation from 220 participants to a target of 302 participants, assuming constant response rates. The trial ultimately recruited 236 participants before recruitment was terminated after the second USM.

The general analytical approach employed suitable regression models, dependent on the underlying data-type, and incorporating repeated responses at all assessment times where possible. Estimates were adjusted for the minimisation parameters and baseline response (where available). If repeated responses were made, models included variables for participant and assessment time (categorical) and to allow for varying treatment effect over time, a time by treatment interaction parameter. All estimates of differences between groups (mean differences or odds ratios) were presented with two-sided confidence intervals. Analysis was conducted for all outcomes for the primary analysis population. Analyses in supportive analysis populations were limited to MMAS scores; amenorrhoea and heavy menstrual bleeding and surgical interventions.

For the primary outcome, following inspection of pooled data as part of data validation processes, a high degree of skew in the responses was considered likely. To account for this, a generalised estimating equation mode^20^ with a cumulative logit link for ordered MMAS scores categorised as ≤50, 51–75, 76–99, = 100 was utilised. These categories have been used previously in similar trials of HMB with MMAS as the primary outcome.^21^ The generalised estimating equating model took into account correlated longitudinal data and a general unstructured covariance matrix was assumed. Cumulative odds ratios and 95% confidence intervals for the treatment group parameter were produced and the statistical significance (p-value) of the treatment group variable determined by an associated chi-squared test.

MMAS scores at three and six months follow-up were analysed as part of the aforementioned model. Bleeding scores from the PBAC were converted into the following categories: i) the proportion with amenorrhoea (=0) and ‘any bleeding’ (score >0) as well as ii) non-heavy (score ≤ 100) and heavy (score>100) bleeding. These outcomes, along with cycle regularity, were analysed in a similar manner to the categorised MMAS scores. Duration of period was another ordinal response and was analysed in a similar manner to the MMAS categorised scores. Data from patient reported outcomes (UFS-QOL, EQ-5D, VAS and SAQ) that returned continuous scores were analysed using linear regression models for repeated measures to estimated mean differences between the two groups at each time point.

Satisfaction and participant rating of treatment were analysed using ordinal logistic regression. Binary clinical observations from the pelvic ultrasound and willing to recommend to a friend were analysed using logistic regression. There were too few events to formally analyse the number of surgical interventions, summary statistics only are presented. The number of serious adverse events was analysed using a chi-squared test. Observations from the endometrial biopsies taken after the end of ulipristal acetate treatment and liver function tests taken during ulipristal acetate courses were tabulated.

### Role of the funding source

The 10.13039/501100000265Medical Research Council and 10.13039/100014461National Institute of Health Research EME Programme (who funded the study) and the study sponsor (10.13039/501100000848University of Edinburgh and NHS Lothian Academic and Clinical Central Office for Research and Development; ACCORD), had no role in study design, data collection, data analysis, data interpretation, writing of the report, or the decision to submit the results for publication. All authors had access to the trial dataset and supported the decision to submit for publication.

## Results

**Fig. 1:**
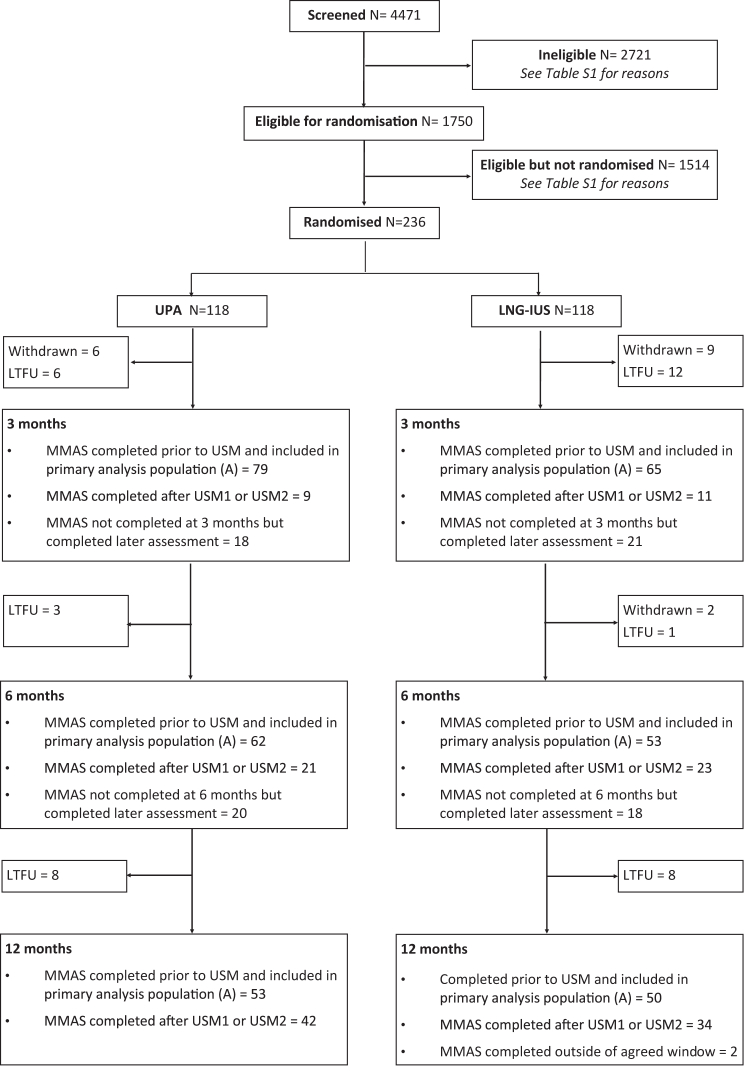
Consort diagram.

The flow of participants through the trial is shown in Fig. 1. 1750 patients were initially considered eligible based on clinical criteria. Of these, 236 were randomised, reasons for non-eligibility and non-randomisation are shown in Table S1. The trial ultimately recruited 236 participants before recruitment was halted after the second urgent safety measure, with 103 participants contributing to the primary analysis population (Fig. 1). MMAS questionnaires were completed at 12 months by 181/236 (77%) of participants. Of the remainder, 17 were formally withdrawn from the trial (the majority at patient request) and 38 were lost to follow-up.

In all randomised participants, the minimisation algorithm ensured balance between groups in terms of age (mean 42.5 years overall), BMI (mean 30.8 kg/m^2^ overall) and the proportion of participants with fibroids (Table 1). Participants contributing to the primary analysis population (Table S2) were similar in nature to the full randomised population (Table 1). In the ulipristal acetate group, 29 participants stopped treatment due to the urgent safety measures and 13 participants ceased treatment for other reasons (Table S3). Seventeen participants had the levonorgestrel-releasing intrauterine system removed prior to 12 months (Fig. S2).

**Table 1:** Baseline characteristics of all randomised participants.

	UPA (N = 118)	LNG-IUS (N = 118)	Overall (N = 236)
Age^a^			
≤35 years	15 (13%)	15 (13%)	30 (13%)
>35 years	103 (87%)	103 (87%)	206 (87%)
Mean (SD)	42.7 (7.0)	42.4 (6.9)	42.5 (7.0)
BMI^a^			
≤25 kg/m^2^	28 (24%)	28 (24%)	56 (24%)
>25 kg/m^2^	90 (76%)	90 (76%)	180 (76%)
Mean (SD)	30.7 (7.0)	30.9 (7.1)	30.8 (7.0)
Duration of symptoms^a^			
<1 year	16 (14%)	12 (10%)	28 (12%)
≥1 year	102 (86%)	106 (90%)	208 (88%)
Median [IQR]	24 [15–64]	48 [15–120], 1	36 [15–84]
Fibroids^a^			
Fibroids>2 cm	31 (26%)	27 (23%)	58 (25%)
Fibroids≤2 cm	12 (10%)	11 (9%)	23 (10%)
No fibroids	75 (64%)	80 (68%)	155 (66%)
Number of fibroids^b^			
1	22 (19%)	21 (18%)	43 (18%)
2	8 (7%)	8 (7%)	16 (7%)
>2	12 (10%)	9 (8%)	21 (9%)
Volume of largest fibroid (ml)^b^			
Median [IQR], n	13.4 [2.9–41.8], 38	8.6 [2.1–40.6], 34	10.5 [2.8–41.2], 72
Missing	5	4	9
Ethnicity			
White	110 (93%)	108 (92%)	218 (92%)
Mixed	2 (2%)	1 (1%)	3 (1%)
Asian	4 (3%)	6 (5%)	10 (4%)
Black	2 (2%)	3 (3%)	5 (2%)
Number of times the patient has been pregnant			
Median [IQR], n	2 [1–3],118	2 [1–3],116	2 [1–3],234
Missing	0	2	2
Result of pregnancy^c^			
Live birth	96 (81%)	86 (73%)	182 (77%)
Still birth	3 (3%)	1 (1%)	4 (2%)
Termination	22 (19%)	17 (14%)	39 (17%)
Miscarriage/ectopic	30 (25%)	20 (17%)	52 (22%)
None reported	3	5	8
Route of deliveries^c^			
Vaginal	73 (62%)	65 (55%)	138 (58%)
Caesarean	29 (25%)	28 (24%)	57 (24%)
Forceps/ventouse	13 (11%)	14 (12%)	27 (11%)
None reported	8	10	18
Previous treatments for HMB c			
Mefenamic Acid/NSAIDs	39 (33%)	39 (33%)	78 (33%)
Tranexamic Acid	71 (60%)	66 (56%)	137 (58%)
Combined Oral Contraceptive	29 (25%)	28 (24%)	57 (24%)
Progesterone Only Pill	21 (18%)	26 (22%)	47 (20%)
Norethisterone	29 (25%)	34 (29%)	63 (27%)
Depo-Provera (medroxyprogesterone acetate)	10 (8%)	5 (4%)	15 (6%)
Implant (Nexplanon/Implanon)	5 (4%)	7 (6%)	12 (5%)
Ulipristal Acetate	0 (−)	1 (1%)	1 (<1%)
LNG-IUS	17 (14%)	16 (14%)	33 (14%)
None reported	0	1	1
Previous Surgical treatments c			
Surgical termination	15 (13%)	10 (8%)	25 (11%)
Surgical management of miscarriage	8 (7%)	5 (4%)	13 (6%)
Uterine curettage	6 (5%)	6 (5%)	12 (5%)
None reported	0	1	1
Evidence of adenomyosis			
Yes	8 (7%)	12 (10%)	20 (8%)
No	92 (78%)	86 (73%)	178 (75%)
Missing	18	20	38

a Minimisation variable.

b Figures based (denominator) on those that were identified as having fibroids on ultrasound.

c More than one option possible.

In the primary analysis population (Table 2), there was no evidence of a difference in MMAS median score category between groups at 12 months: ulipristal acetate group medium score 89, interquartile range [IQR] 65 to 100, versus levonorgestrel-releasing intrauterine system group median score 94, IQR 79 to 100 (adjusted odds ratio [OR] 0.55, 95% confidence interval [CI] 0.26 to 1.17; p = 0.12). The odds of being in a higher MMAS score category, reflecting better quality of life, were higher in the ulipristal acetate group than in the levonorgestrel-releasing intrauterine system group by three months (median: 94, IQR 65 to 100 versus 68, IQR 54 to 94; adjusted OR 2.22, 95% CI 1.24 to 3.96) but this difference was no longer apparent by six months (median: 80, IQR 50 to 100 versus 94, IQR 65 to 100; adjusted OR 0.64, 95% CI 0.33 to 1.24). Estimates of treatment effect were very similar in secondary analysis populations (Tables S4 and S5), were robust to sensitivity analysis (Table S6) and showed no evidence of a varying treatment effect over recruitment period (Table S7).

**Table 2:** MMAS scores in the primary analysis population.

MMAS Category^a^	UPA N (%)	LNG-IUS N (%)	Odds ratio^b^ (95%CI)	p-value
Baseline				
≤50	65 (73%)	54 (68%)		
51-75	16 (18%)	24 (30%)		
76-99	8 (9%)	1 (1%)		
100	–	–		
Median (IQR)	37 [24–51]	33 [24–54]		
TOTAL	89	79		
3 months				
≤50	14 (18%)	16 (25%)	2.22 (1.24, 3.96)	–
51-75	12 (15%)	20 (31%)		
76-99	19 (24%)	17 (26%)		
100	34 (43%)	12 (18%)		
Median (IQR)	94 [65–100]	68 [54–94]		
TOTAL	79	65		
6 months				
≤50	16 (26%)	7 (13%)	0.64 (0.33, 1.24)	–
51-75	12 (19%)	13 (24%)		
76-99	13 (21%)	13 (24%)		
100	21 (34%)	20 (38%)		
Median (IQR)	80 [50–100]	94 [65–100]		
TOTAL	62	53		
12 months^c^				
≤50	12 (23%)	6 (12%)	0.55 (0.26, 1.17)	0.12
51-75	8 (15%)	9 (18%)		
76-99	12 (23%)	12 (24%)		
100	21 (40%)	23 (46%)		
Median (IQR)	89 [65–100]	94 [70–100]		
TOTAL	53	50		

Baseline data included for those in this analysis population and returned a form at either 3, 6 or 12 months.

a Menorrhagia multi-attribute scale questionnaire; score ranges from 0 (severely affected) to 100 (not affected).

b Estimates>1 favour UPA; centre removed from model due to lack of convergence.

c Primary outcome time-point; Number of participants who declined to complete the MMAS on the grounds they are no longer having periods their score will be assumed to be maximum (MMAS = 100): LNG-IUS = 1 (6 months), none at other times.

The proportion of participants experiencing amenorrhoea was much higher in the ulipristal acetate group compared with those in the levonorgestrel-releasing intrauterine system group across all time points (Table 3). Results were similar in both of the secondary analysis populations (Tables S8 and S9). The proportion of participants experiencing heavy bleeding as defined by PBAC score was not noticeably different between groups.

**Table 3:** PBAC bleeding diary scores in the primary population A.

	UPA N (%)	LNG-IUS N (%)	Odds ratio^a^ (95%CI)
Baseline			
Amenorrhoea (=0)	0 (−)	0 (−)	
Light (1–10)	0 (−)	0 (−)	
Normal (>10–100)	4 (5%)	11 (15%)	
Heavy (>100)	75 (95%)	61 (85%)	
Median score [IQR]	306 [173–534]	204 [138–455]	
TOTAL	N = 79	N = 72	
3 Months			
Amenorrhoea (=0)	31 (56%)	3 (5%)	29.3 (7.37, 116)
Light (1–10)	6 (11%)	8 (13%)	
Normal (>10–100)	4 (7%)	32 (50%)	
Heavy (>100)	14 (25%)	21 (33%)	0.64 (0.27, 1.53)
Median score [IQR]	0 [0–199]	53 [21–170]	
TOTAL	N = 55	N = 64	
6 Months			
Amenorrhoea (=0)	20 (53%)	5 (10%)	11.7 (3.78, 36.0)
Light (1–10)	3 (8%)	10 (20%)	
Normal (>10–100)	10 (26%)	29 (57%)	
Heavy (>100)	5 (13%)	7 (14%)	0.83 (0.23, 2.9)
Median score [IQR]	0 [0–37]	22 [7–70]	
TOTAL	N = 38	N = 51	
12 Months			
Amenorrhoea (=0)	18 (64%)	10 (25%)	7.12 (2.29, 22.2)
Light (1–10)	0 (−)	6 (15%)	
Normal (>10–100)	5 (18%)	12 (30%)	
Heavy (>100)	5 (18%)	12 (30%)	0.47 (0.12, 1.79)
Median score [IQR]	0 [0–58]	28 [1–118]	
TOTAL	N = 28	N = 40	

Baseline data included for those in this analysis population and returned a form at either 3, 6 or 12 months.

a Odds Ratio for amenorrhoea (estimates>1 favour UPA) and heavy bleeding (estimates<1 favour UPA) shown; centre removed from model due to lack of convergence; number of participants who declined to complete the Menstrual Blood Loss Diary on the grounds they are no longer having periods, therefore score assumed to be equal to 0: 3 Month (UPA = 13; LNG-IUS = 2); 6 Month (UPA = 12; LNG-IUS = 4); 12 Month (UPA = 9; LNG-IUS = 8).

There was no consistent evidence that the proportion of participants reporting irregular or on-off bleeding was different between the groups (Table S10); similarly menstrual cycle duration was not consistently different between groups across the assessment times (Table S11). There was no evidence of a difference in the other patient reported outcomes (Table S12); the uncertainty around the treatment effect estimates was either too large to rule out no effect or there was lack of consistency across the assessment times.

The number of participants reporting a surgical Intervention was low at 12 months follow-up in all those randomised. Two hysterectomies were reported in the ulipristal acetate group. One participant in the levonorgestrel-releasing intrauterine system group reported having a cystectomy, one a prophylactic bilateral salpingo-oophorectomy and another reporting a ureteric stenting, sigmoidoscopy and biopsy.

No participants treated with ulipristal acetate had evidence of malignancy following endometrial biopsy out to a maximum of 12 months (Fig. S3). Seven participants (8%) had evidence of PRM-associated endometrial changes in their initial biopsy, reducing to one at subsequent biopsy after a further three months and none at three months after that. One participant had a diagnosis of endometrial hyperplasia with atypia.

Following instigation of liver function monitoring after the urgent safety measures (2018 and 2020), two participants of 40 providing blood samples during ulipristal acetate treatment had transaminase levels greater than three times the upper limit of normal, and three of 55 participants in the post-treatment period. Additional participants had results outside of local normal ranges but did not meet the threshold for early discontinuation (Table 4). None of these participants required hospital admission and liver function tests returned to normal in all these participants.

**Table 4:** Liver function tests.

	Numbers of participants (%)	
	At any time during treatment	At any time off-treatment
Number who have had LFT testing^a^	40	55
Number with test result outside local normal range in any test^a^, ^b^	12 (30%)	12 (22%)
Number with clinically significant results in any test^a^, ^b^	2 (5%)	5 (9%)
Number with transaminase levels>3 times upper limit of normal in any test^a^, ^c^	2 (5%)	3 (5%)
Number with both clinically significant and greater than 3 times of upper limit of normal in any test^a^	1 (3%)	3 (5%)

a Numbers provided are per participant (not including multiple participants).

b Aspartate transaminase, alanine transaminase, alkaline phosphatase as a minimum and bilirubin and gamma-glutamyltransferase in some hospitals.

c Transaminase levels>3 times upper limit of normal was the threshold to stop treatment with UPA as advised by the MHRA.

Adverse events categorised using MedDRA coding were varied, with no obvious evidence of a difference between groups (Table S13). There were six serious adverse events in the ulipristal acetate group and five in the levonorgestrel-releasing intrauterine system group (p = 0.76). In the ulipristal acetate group, these were predominantly related to management of unrelated conditions, or, in those using ulipristal acetate for a short duration or had already ceased treatment. In the levonorgestrel-releasing intrauterine system group there were more directly related events, including removal of the system for pelvic pain, complications from previous fibroid surgery or due to concerns about hormonal-related cancers. There was one suspected unexpected serious adverse reaction: one participant with a strong family history of autoimmune hepatitis developed acute hepatitis during the final course of ulipristal acetate and was treated with high dose steroids. This occurred before the introduction of liver function eligibility tests, which would have likely excluded her from trial participation.

## Discussion

Our results suggest that after 12 months, quality of life improved substantially in participants who received either ulipristal acetate or levonorgestrel-releasing intrauterine system, but there was no evidence of a difference between them. Those allocated to ulipristal acetate were more likely to report amenorrhoea at 12 months, although this was not reflected in better quality of life or sexual functioning. There were no malignant changes in the endometrium, with low rates of PRM-associated endometrial changes following ulipristal acetate treatment, which all resolved within five months of ceasing treatment. Only one participant developed endometrial hyperplasia with atypia. Following the introduction of liver function tests for safety, two participants exhibited abnormal liver function, rising to three in the post-treatment period, although none required hospital admission and results returned to the normal range.

In contrast to previous studies of SPRMs, typically limited to those with large fibroids only, the UCON trial involved a wider population affected by HMB: two thirds had structurally normal uteri and greater proportion of participants were white. Good adherence to the ulipristal acetate treatment schedule in those unaffected by the safety measures was observed, with only 13/118 (11%) discontinuing treatment due to perceived lack of efficacy or side effects. This finding was similar to that observed in the PEARL IV trial where 75% completed four 12-week courses of treatment.^22^ Slightly more of those allocated levonorgestrel-releasing intrauterine system (17/118, 14%) discontinued treatment by 12 months, predominantly due to the impact on bleeding patterns, similar to rates observed in the ECLIPSE trial.^7^

The two drug alerts that required urgent safety measures, significantly impacted trial recruitment and processes and are the greatest limitation of this trial. The first urgent safety measure resulted in early cessation of ulipristal acetate treatment for many participants. This impacted both on primary outcome data and the target sample size and introduced a significant level of statistical complexity, whilst the monthly monitoring requirements may also have impacted retention and participant satisfaction. The second safety notice stopped recruitment and all ulipristal acetate use whilst the concurrent Covid-19 pandemic delayed some safety assessments. This resulted in failure to achieve the required sample size to ensure 90% power to address the study hypothesis. A smaller sample size than intended has hampered our ability to detect a conclusive difference in MMAS scores, so we would recommend caution in not interpreting our finding as equivalence for this outcome as the estimates of uncertainty were wide. Inability to blind participants meant we were at increased risk of performance and response bias, but to an unknown extent and direction.

In the sub-set of participants with uterine fibroids, the UFS-QoL health related quality of life domain was similar at three months to that observed in the PEARL II study.^11^ despite participants in UCON having smaller fibroids. Improvements in MMAS scores and EQ-5D utilities for those allocated levonorgestrel-releasing intrauterine system in the UCON trial were broadly similar to those in the ECLIPSE study,^7^ although improvement in EQ-5D visual analogue scale was more marked in the UCON trial (8.6 point improvement) than in the ECLIPSE trial (1.2 point improvement).^7^

In contrast to the group using the levonorgestrel-releasing intrauterine system, those allocated to ulipristal acetate treatment were more likely to report amenorrhoea at 12 months. Amongst UCON participants receiving ulipristal, 64% were amenorrhoeic at the end of the third treatment course, similar to PEARL IV in which 69.6% of those taking 5 mg ulipristal acetate achieved amenorrhoea at the end of their fourth treatment course. However, this did not result in greater improvement in quality of life, reflecting the fact that the impact of HMB and treatment thereof, is more complex that bleeding pattern alone.

The UCON trial is the first randomised controlled trial to assess the use of ulipristal acetate to ameliorate bleeding in those with structurally normal uteruses or small fibroids, and improves quality of life and reduces bleeding in this population. However due to the potential, albeit very rare risk of drug-induced liver injury, ulipristal acetate is only licenced for intermittent treatment of severe symptoms due to uterine fibroids where surgery has either failed or is contraindicated.^1^ The license is unlikely to be extended, despite rates of drug-induced liver injury remaining extremely low, being 11:100,000, a risk comparable or lower than several drugs that are not subject to liver function monitoring and include diclofenac and several antibiotics.^23^ Both risk and impact of quality on life with ulipristal acetate treatment should be considered within the context of alternative treatments for HMB, and limitations thereof. The levonorgestrel-releasing intrauterine system, whilst effective for many may not be suitable or acceptable to all, and alternative hormonal treatments such as GnRH antagonist and agonists may have unwanted side-effects and are associated with more deleterious effect on bone mineral density unless mitigated by concurrent HRT use.^11^^,^^24^^,^^25^ Surgical interventions, particularly hysterectomy, are associated with better quality of life, but are typically fertility ending and carry a higher risk of serious complications, and rates of mortality are markedly higher compared to that of fatal liver injury following UPA treatment (>1:1000 vs 0.1:100,000).^23^ Whilst UPA is now unlikely to be licensed for use in the patient population reflected in the UCON study, SPRMs remain an attractive class of compounds, given their oral route of administration, high rates of amenorrhoea, preservation of bone density and lack of long-term endometrial effects. In the future other SPRMs, that do not have the reactive metabolite formation,^26^^,^^27^ and thereby should be considered as new therapeutic options, given the demonstrable efficacy and acceptability to patients. In particular, those affected by HMB without large fibroids, remain limited in their therapeutic options, particularly when fertility preservation is a priority and the need for effective oral medical treatments for HMB with an acceptable side effect profile remains a research priority and a key unmet need for women across their reproductive life-course.

Both ulipristal acetate and levonorgestrel-releasing intrauterine system improve bleeding symptoms and alleviate the adverse impact of heavy bleeding on quality of life. The findings from this trial support the use of ulipristal acetate in refractory, severe HMB in line with the aforementioned current license. New, effective and acceptable oral medical treatment options are needed to address an important and debilitating unmet clinical need. Future novel pharmacological agents could also include those from the SPRM class with better liver safety profiles, in light of the observed effectiveness and tolerability of ulipristal acetate in the UCON trial.

## Contributors

LHRW, LJM, JPD, ARWW, TJC, MAL, DKH, SB, LS, PS, EPN, NR, SS, RRC, HODC contributed to the design, delivery and interpretation of the trial and secured funding. LJM and VC verified the data and conducted the statistical analysis. LP, SO and CS were responsible for the day-to-day management of the trial. LHRW, LJM, JPD and HODC drafted the report, and all authors provided input into editing for publication and accept responsibility to submit for publication.

## Data sharing statement

All data requests should be submitted to the corresponding author for consideration. Access to anonymised data may be granted after review.

## Declaration of interests

LHRW, LJM, JPD, LP, SO, VC, CES, MAL, PPS, EPN, NR, SIS and JW all have no conflicts of interest to declare. ARWW has received consultancy fees (divided with the University of Edinburgh) from Bayer AG and Mithra. TJC has received honoraria from Gedeon Richter and Orbis. DKH has received grant funding from Wellbeing of Women, MRC and North West Cancer Research, and honoraria from the Canadian Society of Fertility and Andrology. SB receives grant funding from NHS Grampian Endowments Funding and NIHR, and royalties from Cambridge University Press and payment (to University of Aberdeen) for speaking at 11th Singapore International Congress on Obstetrics & Gynaecology, and invited lectures to Merck, Organon a and Ferring. He participates on the METAFOR Data Monitoring Committee and ANODE trial. He is board member of NHS Grampian for which University of Aberdeen receive payment. He receives an honorium from Oxford University Press for his role as Editor in Chief, (Human Reproduction Open) is special Senior Editor Cochrane Gynaecology and Infertility (no honorarium). LS has received grant funding from NIHR and EU Horizon 2020 and has received consultancy fees from Gideon Richter. RRC has been supported as a Clinical Research Fellow by Bayer AG between April 2018 to February 2021. HODC receives grant funding from Biotechnology and Biological Sciences Research Council, grants from Medical Research Council/NIHR to support salaries for research staff & study consumables and a Research collaboration grant from Bayer AG, Berlin with salaries for research staff & study consumables. She has personal receipt of royalties from "Up-To-Date" for an article on Abnormal Uterine Bleeding. She has received consulting fees to the University of Edinburgh from Bayer AG (Consultancy and Scientific Advisory Board advice; paid to institution), Gedeon Richter (Consultancy advice; paid to institution) and Myovant Sciences GmbH (Consultancy and Scientific Advisory Board advice; paid to institution). She has received speaking fees to the University of Edinburgh from Vifor Pharma UK Ltd for speaking at a meeting on abnormal uterine bleeding and iron deficiency anaemia (paid to institution) and travel expenses from SAB (Scientific Advisory Board) in March 2020. She is Chair (from 2022) of the Committee for Menstrual Disorders and Related Health Impacts of the International Federation of Gynecology and Obstetrics (FIGO; no payment received).

## Appendix A Supplementary data

Supplementary material

UCON Study protocol

## References

[1] National Institute for Health and Care Excellence Heavy menstrual bleeding: assessment and management. https://www.nice.org.uk/guidance/ng88.

[2] Shapley M., Jordan K., Croft P.R. (2004). An epidemiological survey of symptoms of menstrual loss in the community. Br J Gen Pract.

[3] Schoep M.E., Nieboer T.E., van der Zanden M., Braat D.D.M., Nap A.W. (2019). The impact of menstrual symptoms on everyday life: a survey among 42,879 women. Am J Obstet Gynecol.

[4] Jain V., Chodankar R.R., Maybin J.A., Critchley H.O.D. (2022). Uterine bleeding: how understanding endometrial physiology underpins menstrual health. Nat Rev Endocrinol.

[5] Munro M.G., Critchley H.O.D., Fraser I.S., Committee F.M.D. (Dec 2018). The two FIGO systems for normal and abnormal uterine bleeding symptoms and classification of causes of abnormal uterine bleeding in the reproductive years: 2018 revisions. Int J Gynaecol Obstet.

[6] Kai J., Dutton B., Vinogradova Y., Hiken N., Gupta J., Daniels J. (2022). Medical treatment for heavy menstrual bleeding in primary care: 10 year observational follow up of the ECLIPSE trial cohort. Health Technology Assessment (HTA).

[7] Gupta J.K., Daniels J.P., Middleton L.J. (2015). A randomised controlled trial of the clinical effectiveness and cost-effectiveness of the levonorgestrel-releasing intrauterine system in primary care against standard treatment for menorrhagia: the ECLIPSE trial. Health Technol Assess.

[8] Bofill Rodriguez M., Bofill Rodriguez M., Lethaby A., Jordan V. (2020). Progestogen-releasing intrauterine systems for heavy menstrual bleeding. Cochrane Libr.

[9] Sangkomkamhang U.S., Lumbiganon P., Laopaiboon M., Mol B.W. (2013). Progestogens or progestogen-releasing intrauterine systems for uterine fibroids. Cochrane Database Syst Rev.

[10] Donnez J., Tatarchuk T.F., Bouchard P. (2012). Ulipristal acetate versus placebo for fibroid treatment before surgery. N Engl J Med.

[11] Donnez J., Tomaszewski J., Vazquez F. (2012). Ulipristal acetate versus leuprolide acetate for uterine fibroids. N Engl J Med.

[12] Gedeon Richter (UK) Ltd (2023). Esmya 5 mg Tablets (ulipristal acetate). https://www.medicines.org.uk/emc/product/3951/smpc#gref.

[13] Medicines and Healthcare products Regulatory Agency MHRA Esmya (ulipristal acetate) and risk of serious liver injury: new restrictions to use and requirements for liver function monitoring before, during, and after treatment. https://www.gov.uk/drug-safety-update/esmya-ulipristal-acetate-and-risk-of-serious-liver-injury-new-restrictions-to-use-and-requirements-for-liver-function-monitoring-before-during-and-after-treatment.

[14] Shaw R.W., Brickley M.R., Evans L., Edwards M.J. (1998). Perceptions of women on the impact of menorrhagia on their health using multi-attribute utility assessment. BJOG An Int J Obstet Gynaecol.

[15] Higham J.M., O'Brien P.M.S., Shaw R.W. (1990). Assessment of menstrual blood loss using a pictorial chart. BJOG An Int J Obstet Gynaecol.

[16] Spies J.B., Coyne K., Guaou Guaou N., Boyle D., Skyrnarz-Murphy K., Gonzalves S.M. (2002). The UFS-QOL, a new disease-specific symptom and health-related quality of life questionnaire for leiomyomata. Obstet Gynecol.

[17] Thirlaway K., Fallowfield L., Cuzick J. (1996). The Sexual Activity Questionnaire: a measure of women's sexual functioning. Qual Life Res.

[18] Herdman M., Gudex C., Lloyd A. (2011). Development and preliminary testing of the new five-level version of EQ-5D (EQ-5D-5L). Qual Life Res.

[19] Norman G.R., Sloan J.A., Wyrwich K.W. (2003). Interpretation of changes in health-related quality of life: the remarkable universality of half a standard deviation. Med Care.

[20] Liang K.Y., Zeger S.L. (1986). Longitudinal data-analysis using generalized linear-models. Biometrika.

[21] Cooper K., Breeman S., Scott N.W. (2019). Laparoscopic supracervical hysterectomy versus endometrial ablation for women with heavy menstrual bleeding (HEALTH): a parallel-group, open-label, randomised controlled trial. Lancet.

[22] Donnez J., Donnez O., Matule D. (2016). Long-term medical management of uterine fibroids with ulipristal acetate. Fertil Steril.

[23] Middelkoop M.A., Bet P.M., Drenth J.P.H., Huirne J.A.F., Hehenkamp W.J.K. (2020). Risk-efficacy balance of ulipristal acetate compared to surgical alternatives. Br J Clin Pharmacol.

[24] Kai J., Middleton L., Daniels J. (2016). Usual medical treatments or levonorgestrel-IUS for women with heavy menstrual bleeding: long-term randomised pragmatic trial in primary care. Br J Gen Pract.

[25] Schlaff W.D., Ackerman R.T., Al-Hendy A. (2020). Elagolix for heavy menstrual bleeding in women with uterine fibroids. N Engl J Med.

[26] Gatti M., Poluzzi E., De Ponti F., Raschi E. (2020). Liver injury with ulipristal acetate: exploring the underlying pharmacological basis. Drug Saf.

[27] Moller C., Bone W., Cleve A. (2018). Discovery of vilaprisan (BAY 1002670): a highly potent and selective progesterone receptor modulator optimized for gynecologic therapies. ChemMedChem.

