# Supplementary material for: Ulipristal acetate versus levonorgestrel-releasing intrauterine system for heavy menstrual bleeding (UCON): a randomised controlled phase III trial
Source: eClinicalMedicine. 2023 May 18;60:101995. doi: 10.1016/j.eclinm.2023.101995 (PMC10209678; doi:10.1016/j.eclinm.2023.101995)

FEBRUARY 2018
RECRUITMENT TO THE UCON TRIAL HAS BEEN SUSPENDED UNTIL FURTHER NOTICE

PLEASE REFER TO THE NOTICE ON PAGE 2.
TRIAL PROTOCOL HAS BEEN UPDATED ACCORDINGLY.

The UCON Trial:

Ulipristal acetate versus conventional management of heavy menstrual bleeding (HMB; including uterine fibroids): a randomised controlled trial and exploration of mechanism of action

PROTOCOL

IMPORTANT NOTICE

Following an Urgent Safety Measure issued by the Co-Sponsors of the UCON trial on 12 February 2018, recruitment to the UCON trial has been suspended until further notice.

An Urgent Safety Measure was put in place following a drug alert for Ulipristal Acetate (UPA) issued on 09 February 2018 by the European Medicine Agency and the MHRA.

The following steps have been taken in line with advice from the competent authority:

1. **Trial recruitment has been suspended**. No further participants will be recruited to the UCON trial until further notice.
2. **Participants currently on a 12 week UPA cycle** should continue with their dose until the end of the 12 week cycle and undergo monthly Liver Function Tests (LFTs) with immediate effect. Subsequent cycle(s) will not be initiated.
3. **Participants currently on a 4 week stopping period** between cycles, should **not** resume UPA.
4. All participants who have **received UPA within the last 30 days** must undergo an LFT at the earliest possibility (ideally within 2 weeks).
5. For any current or recent users of UPA presenting with **signs or symptoms suggestive of liver injury** (e.g. nausea, vomiting, malaise, right hypochondrial pain, anorexia, asthenia, jaundice) – transaminase levels must be checked immediately.
6. **Treatment must be stopped if transaminase levels are more than two times the upper limit of normal** and participants are to be closely monitored and referred for specialist hepatology evaluation if clinically indicated.
7. Participants who stop treatment **should remain** on the trial and be followed up as per the trial protocol.

The protocol and associated documents have been updated to reflect these developments while the EMA review of the safety profile of UPA is ongoing.

Participants who are currently taking UPA or have received a dose in the last 30 days will be contacted by local research teams via telephone to discuss the implications of the Urgent Safety Measure and arrange for LFTs (where appropriate). Local research teams will make every attempt to contact participants by telephone, however, if unavailable, participants will be notified of the alert by letter.

UCON participants who completed UPA treatment >30 days prior to the 12 February 2018 will be contacted by letter to provide detail of the drug alert for UPA.

UCON Trial Management Committee

Chief Investigator

Professor Hilary Critchley
University of Edinburgh
MRC Centre for Reproductive Health
The Queen's Medical Research Institute
47 Little France Crescent
Edinburgh EH16 4TJ
Tel: 0131 242 6858
Email hilary.critchley@ed.ac.uk

Trial Coordination and Statistics

Professor Jane Daniels
University of Nottingham
jane.daniels@nottingham.ac.uk

Mr Lee Priest
University of Birmingham
Email: l.priest.1@bham.ac.uk

Mr Lee Middleton
University of Birmingham
Email: l.j.middleton@bham.ac.uk

Mr Konstantinos Tryposkiadis
University of Birmingham
Email: k.tryposkiadis@bham.ac.uk

Clinical Lead Investigators

Professor Justin Clark
Birmingham Women's Hospital
Tel: 0121 472 1377 Ext 4219
Email: Justin.Clark@bwhct.nhs.uk

Professor Mary Ann Lumsden
University of Glasgow
Tel: 0141 201 8616
Email: maryann.lumsden@glasgow.ac.uk

Dr Dharani Hapangama
University of Liverpool
Tel: 0151 795 9559
Email: dharani.hapangama@liverpool.ac.uk

Dr Lucky Saraswat
University of Aberdeen
Tel: 01224 438 419
Email: lucky.saraswat@nhs.net

Research Fellow (Clinical Co-Investigator)

Dr Paul Smith
Birmingham Women's Hospital
Tel: 07932 044 361
Email: paul.smith@doctors.org.uk

Lead Pathologist

Professor Alistair Williams
University of Edinburgh
Tel: 0131 242 7120
Email: a.williams@ed.ac.uk

Scientific Leads for Mechanistic Studies

Professor Hilary Critchley
University of Edinburgh
Tel: 0131 242 6858
Email: hilary.critchley@ed.ac.uk

Dr Scott Semple
University of Edinburgh
Tel: 0131 242 7757
Email: scott.semple@ed.ac.uk

Professor Neil Roberts
University of Edinburgh
Tel: 0131 242 7769
Email: neil.roberts@ed.ac.uk

Kiaming Yin
University of Edinburgh
Tel: 0131 242 9124
s1158296@sms.ed.ac.uk

Research Collaborators

Dr Michael Thrippleton
University of Edinburgh
Tel: 01315 372 663
Email: m.j.thrippleton@ed.ac.uk

Professor Philippa Saunders
University of Edinburgh
Tel: 0131 242 6388
Email: p.saunders@ed.ac.uk

Dr Pamela Warner
University of Edinburgh
Tel: 0131 650 3248
Email: p.warner@ed.ac.uk

Trial Management Group

Professor Hilary Critchley, University of Edinburgh

Professor Alistair Williams, University of Edinburgh

Professor Neil Roberts, University of Edinburgh

Dr Jane Daniels, University of Birmingham
Dr Max Feltham, University of Birmingham

Mr Lee Priest, University of Birmingham
Miss Emma Barlow, University of Birmingham

Mr Lee Middleton, University of Birmingham

Mr Konstantinos Tryposkiadis, University of Birmingham

Trial Steering Committee (TSC)

Professor Hilary Critchley, University of Edinburgh

Dr Jane Daniels, University of Birmingham

Mr Lee Priest, University of Birmingham (Non-voting member)

Sponsor Representative, University of Edinburgh

Trial Steering Committee (TSC) Independent Members

Prof Ying Cheong (Chair), University of Southampton

Dr Christian Becker, University of Oxford

Dr Emma Crosbie, University of Manchester

Professor Lesley Regan, Imperial College London

Ms Emily O'Toole, PPI Representative

Mr Sanjay Vyas, University of Bristol

Data Monitoring and Ethics Committee (DMEC)

For interim analyses and response to specific concerns

Professor Richard Gray (Chair), Clinical Trial Service Unit and Epidemiological Studies Unit (CTSU), Oxford

Professor Glenn McCluggage, Queen's University of Belfast

Prof Patrick Chien, University of Dundee

UCON Trials Office

Birmingham Clinical Trials Unit (BCTU)
Institute of Applied Health Research
Public Health Building
University of Birmingham
Edgbaston
Birmingham
B15 2TT

Telephone: 0121 414 6665

E-mail: lothian.ucon@nhs.net

Website: www.birmingham.ac.uk/ucon

Twitter: https://twitter.com/UCON_Trial

Trial Coordination

Mr Lee Priest Email: l.priest.1@bham.ac.uk

Miss Emma Barlow Email: e.barlow@bham.ac.uk

Statistics

Mr Lee Middleton Email: l.j.middleton@bham.ac.uk

Mr Konstantinos Tryposkiadis Email: k.tryposkiadis@bham.ac.uk

Database Development

Mr Adrian Wilcockson Email: a.c.wilcockson@bham.ac.uk

Clinical queries should be directed during office hours to the Chief Investigator. Other queries should be directed to the UCON Trials Office.

FOR RANDOMISATIONS

Telephone:	0800 953 0274 (UK)
Website	https://www.trials.bham.ac.uk/UCON

Version Number

Protocol Version 5 – 26 February 2018

Protocol Versions

Version No – Date/ Amendment Type	Summary of Changes Made
5.0 26 February 2018<br><br>Substantial Amendment 08	Following an Urgent Safety Measure (12 February 2018) the following sections of the protocol have been updated accordingly; list of abbreviations; 5.1 Identifying Participants; 5.1.1 Identification from GP databases; 5.1.1.1 Raising awareness of the trial through community pharmacies; 5.1.2 GP Referral to Secondary Care; 5.1.3 Gynaecology Clinic Patient Identification; 5.2 Consenting for eligibility; 5.3 Re-Consenting Participants Following the USM; 5.35.4 Confirmation of eligibility before randomisation/entry into the mechanistic study; 5.5 Ineligible and Non-Recruited participants; 5.6 Randomisation; 5.6.1 Randomisation Procedures; 5.6.2 Treatment Allocation; Figure 2; 5.6.5 Stopping of treatment or Withdrawal of Study Participants; 6.1.7 Dispensing and accountability; 6.2.1 UPA; 6.2.2 LNG-IUS; 6.3 Dose Changes; 6.5 Special and Warning and precautions for use for Ulipristal Acetate (UPA); 7.1 Study Assessment Overview; 7.2 Timing of Study Assessments; Figure 4; Table 1; 10 Adverse Events and Pharmacovigilance; Appendix 1.<br><br>The following new sections have been added; Important Notice (page 2); 5.6.1 Change of Treatment following USM; 6.6.1 Liver Injury (unconfirmed adverse reaction currently under investigation); 8.1.1 Liver Function Test Results.

4.0 – 22nd March 2017	Max Feltham (BCTU Women's Health Team Lead) and Emma Barlow (Data Manager) added. 'Protocol Approval Signatures' - Sponsor signatory confirmed. PI Declaration added. 'Trial Summary' – secondary outcome measures clarified.<br><br>'2.2 RCT Outcomes clarified. '5.1 – Participant Selection and Enrolment' text clarified and new sub section'5.1.1.1 Raising awareness of the trial through community pharmacy' added. "Awareness through Pharmacy" added and clarification of existing pathway to Figure 1. '5.2 Consenting for Screening for Eligibility' – use of pre-screening visit (standard clinical care pathway) ultrasound results added to this section and Figure 2). '5.3 Confirmation of Eligibility before Randomisation/Entry into the Mechanistic Sub-Study. Use of re-screening visit endometrial biopsy results added to this section and Figure 2,3). '5.5 Randomisation' – weeks added to figure 2 and 3 and clarification about use of standard care ultrasound and endometrial biopsy. '5.5.5 Withdrawal of Study Participants' – timing of end of study endometrial biopsy clarified. '6.1.8 Summary of Product Characteristics' – reference to SmPC Reference Document. '6.2.2 LNG-IUS – text clarified. 6.7.1 Fitting of the LNG-IUS – text clarified. '7.3 Outcomes Collected at Study Assessments updated and Table 1 updated. '8 Data Collection – elements of participant questionnaire clarified. '8.1.7 End of Study Visit Form' section added and corresponding section 8.2 updated. 'Section 8.4 Quality Assurance of Endometrial Biopsy Assessments'. 10.4.2 Assessment of Causality' - reference to SmPC Reference Document.
3.0 – 25th January 2016/ Substantial Amendment (AM3)	MRC logo added, list of abbreviations updated, change of BCTU staff.<br><br>Trial Summary updated ('at least 5 NHS hospitals')<br><br>'1.1 Background' – reference to Mirena removed. '2.2.2 Secondary Outcomes' updated. '4.2.1 Inclusion criteria' – bleeding 'at intervals of 21 – 42 days removed'. '5.1.3 Gynaecology Clinic Patient Identification' – figure 1 updated to clarify that blood samples are to observe serum haemoglobin and oestradiol levels; '5.5.5 Withdrawal of Study Participants' – clarification of duration LNG-IUS may be used depending on manufacturer. '6.1.3 Levonorgestrel (Reference)' – clarification that in context of trial, of LNG-IUS may be manufactured by two companies. '6.1.5 Marketing Authorisation Holder' – marketing authorisation codes for Levosert added. '6.3 Dose Changes' – acceptable timeframe for women participant start taking UPA. '6.4.2 Monitoring Compliance' – Clarification of drug compliance. '7.2 Timing of Study Assessments' clarification of menstrual blood loss diary completion. '7.3 Outcomes Collected at Study Assessments' – clarification that MRI taken at Edinburgh will be performed in final week of treatment. '8.1.1 Participant Questionnaire' – EuroQol/ ICECAP added. '8.1.3 Clinical Assessment and Randomisation Form' – clarification of serum blood levels observed. '9.2.4 Handling missing data and other sensitivity analysis – clarification of follow-up.

2.0 – 30th June 2015/ Substantial Amendment (AM2)	Admin changes - trial logo added, TSC/ UCON Trials Office Updated, trial registration numbers updated.<br><br>Trial Summary – eligibility updated.<br><br>‘2.2 - RCT Outcomes’ section updated with surgical intervention. ‘3.1 Design’ clarified. ‘4.1 number of participants’ clarified. ‘4.2 – Eligibility’ clarified. ‘5 Participant Selection and enrolment’. Figure 1 updated. ‘5.2 Consenting for Screening for Eligibility’ clarified. ‘5.5 Randomisation’ clarified. Figure summarising patient pathway for Edinburgh patients inserted. ‘6 – Investigational Medicinal Products’ clarified. ‘7 Study Assessments Overview’ clarified. ‘8 – Data Collection’ clarified. ‘9 – Statistics and Data Analysis’. ’10 – Adverse Events and Pharmacovigilance’. ’11 - Trial Management and Oversight Arrangements’ admin changes.

Trial Registration Numbers

Sponsor	University of Edinburgh and NHS Lothian
Sponsor Reference Number	UCON
Funder	Medical Research Council (MRC) and National Institute for Health Research (NIHR) - Efficacy and Mechanism Evaluation (EME)
Funding Reference Number	12/206/52
Chief Investigator	Professor Hilary Critchley
Sponsor Representative	Dr Vikki Young
EudraCT Number	2014-003408-65
REC Number	14/LO/1602
MHRA CTA Reference Number	275860016
ISRCTN Number	20426843
UKCRN Portfolio ID	18534
University of Birmingham Ref	ERN_14-0938
IRAS Project ID	145282
NHS Research Scotland (NRS) Number	15-GY30

PROTOCOL APPROVAL SIGNATURES

The investigators and the sponsor have discussed this protocol. The investigators agree to perform the investigation and to abide by this protocol except in case of medical emergency or where departures from it are mutually agreed in writing.

This protocol was written in accordance with the sponsor's procedures available at:
http://www.accord.ed.ac.uk/standardopprocs/CRSOPs.html

Chief Investigator

Professor Hilary Critchley 28 Mar 2018

University of Edinburgh Signature Date

Trial Statistician

Mr Lee Middleton 28 Mar 2018

University of Birmingham Signature Date

Clinical Research Facilitator, Co-Sponsor's Representative

Dr Fiach O'Mahony 28 March 2018

University of Edinburg Signature *Date*

Principal Investigator's Declaration

Site: ____

Principal Investigator: ____

Trial Name: Ulipristal acetate versus conventional management of heavy menstrual bleeding (HMB; including uterine fibroids): a randomised controlled trial and exploration of mechanism of action

I confirm I have received, read and understood the aforementioned version of the trial protocol. I confirm my team and I will adhere to this version of the protocol following receipt of the required local approvals.

Signature: ____

Date: D D - M M M - Y Y Y Y

The Principal Investigator should sign this page and email a copy of this page to the

UCON Trial Office via lothian.ucon@nhs.net

LIST OF ABBREVIATIONS

ACCORD	Academic and Clinical Central Office for Research & Development - Joint office for University of Edinburgh and NHS Lothian
AE	Adverse Event
AR	Adverse Reaction
BCTU	Birmingham Clinical Trials Unit
CI	Chief Investigator
CRF	Case Report Form
DMEC	Data Monitoring and Ethics Committee
EudraCT	European Clinical Trials Database
EQ-5D-5L	EuroQol-5 Dimension-5 Level (Quality of Life Questionnaire)
GCP	Good Clinical Practice
ICECAP-A	ICEpop CAPability measure for Adults (Quality of Life Questionnaire)
ICH	International Conference on Harmonisation
IMP	Investigational Medicinal Product
ISF	Investigator Site File
ISRCTN	International Standard Randomised Controlled Trial Number
LFT	Liver Function Test
LNG-IUS	Levonorgestrel releasing intra-uterine system
MHRA	Medicines and Healthcare Products Regularity Authority
MRI	Magnetic Resonance Imaging
NIHR	National Institute for Health Research
SAE	Serious Adverse Event
SAR	Serious Adverse Reaction
SmPC	Summary of Product Characteristics
SOP	Standard Operating Procedure
SUSAR	Suspected Unexpected Serious Adverse Reaction
TMG	Trial Management Group
TMF	Trial Master File
TSC	Trial Steering Committee
UAR	Unexpected Adverse Reaction
UPA	Ulipristal Acetate
USM	Urgent Safety Measure
UFS-QoL	Uterine Fibroid Symptom-Quality of Life

CONTENTS

CONTENTS .... **xiv**

TRIAL SUMMARY **17**

1 INTRODUCTION **21**

1.1 BACKGROUND 21

1.2 Risks and benefits 22

1.3 RATIONALE FOR STUDY 22

2 STUDY OBJECTIVES **22**

2.1 RCT OBJECTIVES 22

2.2 RCT OUTCOMES 23

2.3 Functional and mechanistic outcomes 24

3 STUDY DESIGN **24**

3.1 DESIGN 24

4 STUDY POPULATION **24**

4.1 NUMBER OF PARTICIPANTS 25

4.2 ELIGIBILITY 25

4.3 CO-ENROLMENT 26

5 PARTICIPANT SELECTION AND ENROLMENT **27**

5.1 Identifying Participants 27

5.2 CONSENTING FOR SCREENING FOR ELIGIBILITY 30

5.3 confirmation of eligibility before randomisation/ ENTRY INTO THE MECHANISTIC SUB-STUDY 31

5.4 INELIGIBLE AND NON-RECRUITED PARTICIPANTS 31

5.5 RANDOMISATION 31

6 INVESTIGATIONAL MEDICINAL PRODUCT(S) **37**

6.1 STUDY DRUG 37

6.2 DOSE AND DELIVERY OF IMPS 39

6.3 DOSE CHANGES 40

6.4 PARTICIPANT COMPLIANCE 40

6.5 SPECIAL WARNINGS AND PRECAUTIONS FOR USE for ulipristal acetate 40

6.6 Known adverse reactions for Ulipristal Acetate 42

6.7 SPECIAL WARNINGS AND PRECAUTIONS FOR USE for levonorgestrel 42

6.8 Known adverse REACTIONS To levonorgestrel 43

6.9 OTHER MEDICATIONS 44

7 STUDY ASSESSMENTS **44**

7.1 study assessments overview 44

7.2 Timing of study assessments 45

7.3 outcomes collected at STUDY ASSESSMENTS 47

8 DATA COLLECTION 48

8.1 data collection forms 48

8.2 Source data 50

8.3 data management 50

8.4 QUALITY ASSURANCE OF ENDOMETRIAL BIOPSY ASSESSMENTS 51

8.5 Mechanistic Sub-study 51

9 STATISTICS AND DATA ANALYSIS 53

9.1 SAMPLE SIZE CALCULATION 53

9.2 PROPOSED ANALYSES - RCT 53

10 ADVERSE EVENTS AND PHARMACOVIGILANCE 55

10.1 DEFINITIONS 55

10.2 IDENTIFYING AEs AND SAEs 56

10.3 RECORDING AEs AND SAEs 57

10.4 ASSESSMENT OF AEs AND SAEs 57

10.5 REPORTING OF SAEs/SARs/SUSARs 58

10.6 REGULATORY REPORTING REQUIREMENTS 59

10.7 FOLLOW UP PROCEDURES 59

10.8 PREGNANCY 59

11 TRIAL MANAGEMENT AND OVERSIGHT ARRANGEMENTS 60

11.1 TRIAL MANAGEMENT GROUP 60

11.2 The UCON Trial Office 60

11.3 TRIAL STEERING COMMITTEE 60

11.4 DATA MONITORING COMMITTEE 61

11.5 INSPECTION OF RECORDS 61

11.6 RISK ASSESSMENT 61

11.7 STUDY MONITORING AND AUDIT 62

12 GOOD CLINICAL PRACTICE 62

12.1 ETHICAL CONDUCT 62

12.2 REGULATORY COMPLIANCE 62

12.3 INVESTIGATOR RESPONSIBILITIES 62

13 STUDY CONDUCT RESPONSIBILITIES 65

13.1 PROTOCOL AMENDMENTS 65

13.2 PROTOCOL VIOLATIONS AND DEVIATIONS 65

13.3 SERIOUS BREACH REQUIREMENTS 65

13.4 STUDY RECORD RETENTION 65

13.5 END OF STUDY 66

13.6 CONTINUATION OF DRUG FOLLOWING THE END OF STUDY 66
13.7 INSURANCE AND INDEMNITY 66
14 REPORTING, PUBLICATIONS AND NOTIFICATION OF RESULTS 67
14.1 AUTHORSHIP POLICY 67
14.2 PUBLICATION 67
15 REFERENCES 68
16 Appendix 1: TRIAL SCHEMA 70

TRIAL SUMMARY

DESIGN:	A multicentre, randomised controlled trial with an embedded mechanistic evaluation of UPA compared to LNG-IUS.
SETTING:	At least 5 NHS hospitals within the United Kingdom
TARGET POPULATION:	Women aged over 18 years or over, who are presenting to primary and/or secondary care with HMB.<br><br>Exclusion criteria:<br><br>Post-menopausal, a uterus >14 week fibroid uterus and/or cavity length >11cm, submucosal fibroids >2cm in diameter, contraindications to UPA or LNG-IUS; intention to continue current use of CYP3A4 inhibitors (e.g. erythromycin propionate; ketoconazole); intention to continue current use of CYP3A4 inducers (Phenytoin, carbamazepine, rifampicin, St John's Wort), intention to continue current use of P-glycoprotein substrate (e.g. digoxin); past, current or suspected diagnosis of endometrial hyperplasia or neoplasia, severe hepatic impairment; Epilepsy managed with carbamazepine, phenytoin; significant renal impairment; pregnant; current plans to become pregnant within 12 months; currently breastfeeding, severe asthma that is not sufficiently controlled by oral glucocorticoids; past or current known history of uterine, cervical, ovarian or breast cancer; receiving P-glycoprotein substrates; current use progestagen-releasing intrauterine device (except if allocated within UCON), intention to continue continued regular use of Mefenamic acid, intention to continue intention to continue regular use of Mefenamic acid, intention to continue regular use of GnRH analogues, continued regular use of Progestagen-only contraceptive, intention to continue continued regular use of combined oral contraceptive pills, intention to continue hormonal replacement therapy.

HEALTH TECHNOLOGIES<br><br>ASSESSED:	Those allocated to UPA will receive proprietary ulipristal acetate 5mg, orally, once daily. The participant should start taking UPA within the first five days of starting menstrual bleeding.<br><br>Women will be instructed to take UPA in 3 courses according to the following cyclical regime (± 5 days):<br><br>1. One 5mg tablet of UPA to be taken daily for 12 weeks then stopped for 4 weeks, when light vaginal bleeding may occur (withdrawal bleed).<br><br>2. After 4 weeks off treatment, regardless of whether they experience a withdrawal bleed, they should recommence UPA 5mg daily for another 12 weeks, then stop for 4 weeks, when they will expect to have a withdrawal bleed.<br><br>3. Repeat as for treatment course 2.<br><br>OR<br><br>Levonorgestrel-releasing intra-uterine system, retained for up to 5 years (depending on the product and manufacturer).<br><br>Where contraception is required, the woman will be asked to use a barrier method.

OUTCOME MEASURES:	Primary Outcome:<br>The primary outcome measure is the condition-specific Menorrhagia Multi-Attribute Scale (MMAS) designed and validated to capture the impact of HMB on women's day-to-day life (1). HMB is a subjective problem and quality of life is affected by practical difficulties and the impact on social life, psychological wellbeing, physical health, work routine and family life. The menorrhagia multi-attribute utility assessment (MMAS)<br>questionnaire attempts to capture the consequences of HMB on these domains with 6 questions each with 4 levels of response. Summary scores range from 0 (not affected) to 100 (worst affected). The primary time point for analysis will be at 12 months.<br>Secondary Outcome measures:<br>• Menstrual bleeding will be captured by validated Pictorial Blood Loss Assessment Chart (PBAC) (2). The standard PBAC is a validated and well used assessment of menstrual blood loss in women. It will be used to generate the incidence of amenorrhoea, light, normal and heavy menstrual bleeding. It will also be used to ascertain the number of days bleeding per month.<br>• Cycle regularity and duration.<br>• Visual analogue scales (0-10) for pelvic pain during periods, intercourse and at other times.<br>• Uterine Fibroid Symptom and Quality of Life (UFS-QoL) instrument, which contains a health related quality of life (HRQoL) domain and a symptom domain (3). This instrument will be only given to women diagnosed with fibroids.<br>• Sexual Activity Questionnaire (4), a measure of sexual functioning, used in other HMB trials. The sexual activity questionnaire is a valid, reliable and acceptable measure for describing the sexual functioning of women in terms of pleasure and discomfort. It is quick and easy to administer and has good face validity delineating between the sexual functioning of pre and post-menopausal women.<br>• Generic health-related quality of life (HRQOL) will be assessed using the EuroQol EQ-5D-5L instrument, and used to calculate utilities (5) We will also collect data on women's capabilities, as measure of wellbeing using the 5 question ICECAP-A instrument (6).<br>• Satisfaction with treatment outcome measured on a 5-point Likert scale.<br>• Participant rating of effect of treatment on HMB over 12 months measured on a 4-point Likert scale.<br>• Whether participant is willing to recommend the treatment to a friend.<br>• Surgical intervention (hysterectomy, endometrial ablation and other gynaecological surgery)<br>• Adherence to trial treatments and reasons for changing treatment, as reported by the participant.<br>• Serious adverse events and reactions reported by participants, principally those that are serious and detailed in the respective Summary of Product Characteristics (SmPC) and those that are unexpected.

	• Clinical measurements to assess safety and efficacy will include pelvic ultrasound (endometrial appearance; fibroid volume, presence of fibroids) and endometrial biopsies (reported according to pre-agreed criteria by independent pathologists blinded to treatment allocations). Blood samples will be taken to observe serum haemoglobin and oestradiol levels.<br>• Clinical measurement via endometrial biopsy: primary diagnosis (normal/benign/hyperplasia/malignant) and further sub-diagnoses if non-normal.<br>• Clinical measurement via blood samples: serum haemoglobin and oestradiol levels.<br><br>Functional and mechanistic outcomes<br>• Impact on endometrial tissue architecture including regulation of the vascular compartment<br>• Impact on endometrial steroid responsiveness, proliferation, cell survival and inflammatory processes<br>• Expression of genes implicated in pre-malignant change including tumour suppressors<br>• Effects on uterine/ fibroid structure and vascularity as determined by MRI-DCE and high resolution structural MRI
ANALYSIS:	Analysis of the primary outcome will be performed using a linear regression model to estimate differences in MMAS responses between groups at each time-point, including baseline score and the minimisation variables as covariates. Twelve months will be considered the primary outcome time. Point estimates, 95% confidence intervals and p-values from two-sided tests will be calculated for all main outcome measures.<br><br>The mechanistic sub-study will investigate possible changes in defined histological, immunological and molecular/ cellular parameters within treatment groups over time and will be analysed using paired t-tests following an appropriate transformation and also by using non-parametric methods (Wilcoxon signed rank test) for confirmation.
SAMPLE SIZE:	The trial has been designed to detect a 13 points difference in MMAS score between the two groups at 12 months. To detect this size of difference (approximately 0.5 standard deviations) with 90% power (p=0.05) will require 86 women per group, 172 in total. To allow for a 20% loss to follow-up or pregnancy, the sample size has been inflated to 220 women in total.<br><br>In the mechanistic evaluation (performed at the Royal Infirmary of Edinburgh only), a sample size of 20 would give >90% power (p=0.05) to detect a change from baseline – for example in PH3 immunoscore in the endometrium of >1 standard deviation.

1 INTRODUCTION

1.1 BACKGROUND

Menstrual bleeding complaints affect quality of life and comprise a substantial societal burden, including major impact on health care use and costs. In the UK, 1 million women annually seek help for heavy menstrual bleeding (HMB; Clinical Guideline 44; http://www.nice.org.uk/guidance/cg44) and reported treatment costs exceed £65m; an estimated 3.5 million work-days are lost annually (7).

Current commonly prescribed medical treatments for HMB include COX-inhibitors, anti-fibrinolytic therapy, and the levonorgestrel-releasing intra-uterine system (LNG-IUS). NICE recommends LNG-IUS as the first line medical treatment. The LNG-IUS significantly reduces the burden of heavy menstrual bleed compared to non-hormonal treatments, substantially reduces menstrual blood loss, often resulting in amenorrhoea, but the unpredictable unscheduled bleeding may be problematic, with up to a third ceasing use within 2 years (8, 9).

A new group of pharmacological agents, called selective progesterone receptor modulators (SPRMs) are in development and have the potential to provide effective oral treatment for HMB. These SPRMs impart a tissue-specific partial progesterone antagonist effect, acting on progesterone receptors in both endometrial and underlying myometrial tissue. Ulipristal Acetate (UPA) is the only SPRM to have been licensed for use in clinical practice albeit restricted to two cycles of 3 month pre-treatment of fibroids prior to surgical removal. The introduction of this drug followed evaluation in two concurrent randomised controlled trials (RCTs) (10); 'PEARL I' assessed the efficacy of UPA 5mg and 10 mg daily on uterine bleeding and fibroid volume against placebo and 'PEARL II' assessed the efficacy and side effects of UPA versus the gonadotrophin-releasing hormone analogue (GnRHa) leuprolide acetate for treating symptomatic uterine fibroids prior to surgery (10). Both trials demonstrated control of HMB in over 90% of women and amenorrhoea in over 70% women. Control of HMB was achieved significantly more quickly in the UPA group. There was a statistically significant reduction in uterine fibroid size (-21% in the 5mg and -12% in the 10mg groups). Compliance with treatment over 3 months was high in both studies (96% and 98%) and reported side-effects were limited to minor complaints, of which headache (4%) and breast complaints (4%) were the most common, with no difference between active drug and placebo.

Different classes of SPRM induce distinct endometrial morphology, which can be confused with complex hyperplasia. To date detailed analysis of endometrial histology has been limited to treatment with UPA for 3 months (11, 12); detailed histological evaluation showed altered architectural glandular features including extensive cystic dilatation. The glandular epithelium appeared inactive or contained abortive subnuclear vacuolization, occasional mitoses, and apoptosis. Histology returned to normal after discontinuation of treatment (12). Treatment of monkeys for 39 weeks revealed similar endometrial histology to that in women (13).

PTEN is a tumour suppressor gene product, described as a gatekeeper for initiation of carcinogenesis in the endometrium (14, 15). Loss of PTEN function occurs as an early event in endometrial carcinogenesis and has been proposed as a biomarker for premalignant disease even in histologically normal endometrium (14, 16). Progesterone plays an important role in eliminating PTEN-deficient endometrial cells when administered via a progestin-releasing intrauterine device (17) or systemically (18). A compound with progesterone antagonist activity, such as UPA, may raise concerns of an unfavourable effect on PTEN expression and thus on the potential to influence predisposition to latent endometrial precancerous lesions. Hence study of PTEN in women administered UPA is important.

1.2 RISKS AND BENEFITS

Whilst short term use of UPA has been shown to be effective in treating HMB associated with uterine fibroids (3-10cm in size), UPA has the potential to provide a safe, fertility preserving, rapidly effective and convenient oral medical treatment, suitable for women with HMB throughout reproductive age whether associated with fibroids or not. However, whilst UPA has the potential to revolutionise the treatment of HMB, our understanding of the mechanism and location of action of UPA is unclear, as is its longer term safety and effectiveness. As with earlier SPRMs, UPA induces non-physiological endometrial changes known as progesterone-receptor modulator-associated endometrial changes (PAECs) in 62% of participants receiving 5mg UPA, although there is no evidence these are premalignant. Whilst these changes are reported to be reversed in all women after 6 months of ceasing treatment, the mechanisms underlying these changes and their clinical significance remain uncertain. More recent unpublished data provide further reassurance of the reversibility of PAEC - if endometrial biopsy is performed after one normal menstrual shedding after treatment withdrawal, the incidence of PAEC is reduced to around 30%, a rate that remains similar after up to 4 UPA treatment cycles (Personal communication A Williams).

1.3 RATIONALE FOR STUDY

The rationale for using UPA to control HMB is because HMB is a clinical area of unmet need, with a community prevalence of 25%, and can significantly impact on women's lives and burden individuals and healthcare systems. HMB often co-exists with uterine fibroids, benign tumours of uterine muscle present in up to 80% of women of reproductive age. Medical therapy for HMB, particularly when fibroids are present, may be either ineffective or associated with unacceptable side effects. Preservation of fertility is an issue for many women, given the trend for later births.

It is clear that there is an urgent need to develop safe, simple, acceptable, fertility-sparing medical treatments for HMB. SPRMs may provide a solution in light of the mounting evidence that progesterone and the progesterone receptor play a pivotal role in both menstruation and fibroid growth and development. The PEARL studies demonstrated control of HMB in over 90% of women and amenorrhoea in over 70% women. There were no serious side effects or complications associated with UPA; adverse events were limited to minor complaints. However despite profound therapeutic potential, robust data on long term effectiveness and the mechanisms of action of SPRMs in women with HMB remain to be elucidated. There is an urgent need to evaluate the use of UPA against current best medical treatment for all women with HMB.

2 STUDY OBJECTIVES

2.1 RCT OBJECTIVES

2.1.1 Primary Objective

- Determine if UPA is more effective at reducing the burden of HMB symptoms than LNG-IUS after 12 months of treatment.

2.1.2 Secondary Objectives

- Ascertain whether UPA use beyond 3 months and up to 12 months duration is associated with histological changes to the endometrium, and if so, whether this compromises safety.

- Ascertain whether UPA is more effective than LNG-IUS in relation to menstrual blood loss, sexual activity, generic quality of life, satisfaction with treatment, patient reported adverse events, and compliance at 3, 6 and 12 months.
- Determine the response to UPA and LNG-IUS treatment difference in the presence of uterine fibroids in terms of (i) alleviation of HMB and (ii) change in uterine/fibroid volume.

2.1.3 Mechanistic Sub-study Objectives

To understand how UPA causes a reduction in menstrual bleeding and uterine/ fibroid volume in women with HMB, we will determine whether:

- Administration of UPA alters endometrial cell function (proliferation, apoptosis, expression of steroid receptors, tumour suppressors or inflammatory mediators).
- UPA reduces blood flow and blood volume in the endometrium, junctional zone, outer myometrium and fibroid tissue.
- UPA alters the volume fraction of the extracellular matrix in the above tissues.
- UPA reduces uterine and fibroid volume.

2.2 RCT OUTCOMES

2.2.1 Primary outcome

The primary outcome measure is the condition-specific Menorrhagia Multi-Attribute Scale (MMAS) designed and validated to capture the impact of HMB on women's day-to-day life (19). HMB is a subjective problem and quality of life is affected by practical difficulties and the impact on social life, psychological wellbeing, physical health, work routine and family life. The menorrhagia multi-attribute utility assessment (MMAS) questionnaire attempts to capture the consequences of HMB on these domains with 6 questions each with 4 levels of response. Summary scores range from 0 (not affected) to 100 (worst affected). The primary time point for analysis will be at 12 months.

2.2.2 Secondary outcomes

- Menstrual bleeding will be captured by validated Pictorial Blood Loss Assessment Chart (PBAC) (20). The standard PBAC is a validated and well used assessment of menstrual blood loss in women. It will be used to generate the incidence of amenorrhoea, light, normal and heavy menstrual bleeding. It will also be used to ascertain the number of days bleeding per month.
- Cycle regularity and duration.
- Visual analogue scales (0-10) for pelvic pain during periods, intercourse and at other times
- Uterine Fibroid Symptom and Quality of Life (UFS-QoL) instrument, which contains a health related quality of life (HRQoL) domain and a symptom domain (21). Sexual Activity Questionnaire (22), a measure of sexual functioning, used in other HMB trials (9). The sexual activity questionnaire is a valid, reliable and acceptable measure for describing the sexual functioning of women in terms of pleasure and discomfort. It is quick and easy to administer and has good face validity delineating between the sexual functioning of pre and post-menopausal women.

- General quality of life questionnaires - EuroQoL-5D (EQ-5D-5L) (23) and ICECAP-A (24).
- Satisfaction with treatment outcome measured on a 5-point Likert scale.
- Participant rating of effect of treatment on HMB over 12 months measured on a 4-point Likert scale.
- Whether participant is willing to recommend the treatment to a friend.
- Surgical intervention (hysterectomy, endometrial ablation and other gynaecological surgery)
- Adherence to trial treatments and reasons for changing treatment, as reported by the participant.
- Serious adverse events and reactions reported by participants, principally those that are serious and detailed in the respective Summary of Product Characteristics (SmPC) and those that are unexpected.
- Clinical measurements via pelvic ultrasound: uterine volume, evidence of adenomyosis, presence of fibroids, largest fibroid volume, endometrial thickness, endometrial appearance (regular/irregular), evidence of ovarian cysts.
- Clinical measurement via endometrial biopsy: primary diagnosis (normal/benign/hyperplasia/malignant) and further sub-diagnoses if non-normal.
- Clinical measurement via blood samples: serum haemoglobin and oestradiol levels.

2.3 FUNCTIONAL AND MECHANISTIC OUTCOMES

- Impact on endometrial tissue architecture including regulation of the vascular compartment
- Impact on endometrial steroid responsiveness, proliferation, survival and inflammatory processes
- Expression of genes implicated in pre-malignant change including tumour suppressors
- Effects on uterine/ fibroid structure and vascularity as determined by MRI-DCE and high resolution structural MRI

3 STUDY DESIGN

3.1 DESIGN

A multicentre, randomised controlled trial of UPA compared to LNG-IUS with a concurrent mechanistic evaluation of UPA. A trial schema is shown in Appendix 2.

4 STUDY POPULATION

The target population is women who present to primary and secondary care with HMB. Participants will be recruited from the gynaecological, out-patient clinics of participating centres, fitting around their current service provision. Recruitment will be supported by dedicated research nurses, who will work with local gynaecology leads. A flowchart of the recruitment process is shown in Figure 1.

4.1 NUMBER OF PARTICIPANTS

UCON will aim to recruit a minimum of 220 women into the randomised controlled trial and from those, approximately 20 women will be required to undergo more detailed evaluation to contribute to the mechanistic study which is conducted at the Royal Infirmary of Edinburgh. The statistical basis of the sample size calculation is detailed in Section 9.1.

Recruitment will take place over 24 months from 5 centres (i.e. 2 participants/ centre/month).

4.2 ELIGIBILITY

In order to be randomised into the UCON trial, all eligibility criteria must be satisfied. Investigators will be asked to confirm each eligibility criteria at randomisation.

4.2.1 Inclusion Criteria

- Aged 18 years or over
- Menstrual bleeding that she perceives to be heavy and troublesome
- Willing to receive medical treatment with either UPA or LNG-IUS
- Willing to undergo two pelvic ultrasounds
- If allocated to UPA, willing and eligible to undergo two endometrial biopsies with the possibility of a third and fourth (i.e. up to four biopsies)
- If allocated to UPA mechanistic sub-study, willing and eligible to undergo three endometrial biopsies with the possibility of a fourth and fifth (i.e. up to five biopsies). If 'No' may be randomised to RCT if UPA endometrial biopsy consent given
- Willing to use barrier contraception if allocated to UPA
- Given written informed consent
- Willing and eligible to undergo up to three magnetic resonance imaging scans? If allocated to UPA, mechanistic sub-study only. If 'No' may still be randomised to RCT

4.2.2 Exclusion Criteria

- Post-menopausal
- A >14 week fibroid uterus and/or cavity length >11 cm confirmed by ultrasound scan
- Submucosal fibroids >2cm diameter confirmed by ultrasound scan
- Contraindications to UPA or LNG-IUS
- Intention to continue current use of Cytochrome P450 (CYP3A4) inhibitors
- Intention to continue current use of Cytochrome P450 (CYP3A4) inducers (e.g. Phenytoin, carbamazepine, rifampicin, St John's Wort)

- Intention to continue current use of P-glycoprotein substrates (e.g. digoxin)
- A past, current or suspected diagnosis of endometrial hyperplasia or neoplasia
- Severe hepatic impairment
- Epilepsy managed with carbamazepine, phenytoin
- Significant renal impairment
- Pregnant
- Current plans to become pregnant within 12 months
- Currently breastfeeding
- Severe asthma that is not sufficiently controlled by oral glucocorticoids
- Past or current known history of with uterine, cervical, ovarian or breast cancer.
- Current use of progestagen-releasing intrauterine device (except if allocated within UCON)
- Intention to continue regular use of Mefenamic acid
- Intention to continue regular use of Tranexamic acid
- Intention to continue regular use of GnRH analogues
- Intention to continue regular use of Progestagen-only contraceptive
- Intention to continue regular use of any combined oral contraceptive pills
- Intention to continue regular use of hormonal replacement therapy

4.2.3 Exclusion of particular populations

Renal impairment is not expected to significantly alter the elimination of UPA. In the absence of specific studies, UPA is not recommended for patients with severe renal impairment unless the patient is closely monitored.

There is no therapeutic experience with UPA in patients with hepatic impairment, which is expected to alter the elimination of UPA, resulting in increased exposure. This is considered not to be clinically relevant for patients with mildly impaired liver function. UPA is not recommended for use in patients with moderate or severe hepatic impairment unless the patient is closely monitored.

UPA use in women with severe asthma insufficiently controlled by oral glucocorticoids is not recommended.

4.3 CO-ENROLMENT

Women randomised to the UCON trial should be excluded from participation in any further trial of investigational medicinal products (IMPs) for the treatment of gynaecological disorders or infertility. If the woman has withdrawn from the trial treatment but is still contributing to data collection, any further treatments within trials for her HMB should be noted, for example if she chooses to participate in a trial of surgery for HMB.

Women already participating in another trial of an IMP for a non-gynaecological reason are able to participate in UCON, provided careful consideration of the interactions between that IMP and the

UCON trial treatments is undertaken. Arrangements for co-enrolment with another CTIMP will be bound by a written agreement between the Chief Investigator and Co-Sponsors of both/all CTIMPs implicated. This agreement will include special safety reporting measures if required; a minimum wash-out period between last dose in one study and first dose in another; a statement to indicate that the chairs of the TSC/DMC from each study and statisticians form each study that they have no objections to the proposals for co-enrolment; and a statement that arrangements for attribution of liability for co-enrolled participants have been put in place agreed between the sponsors of both/all CTIMPs implicated.

5 PARTICIPANT SELECTION AND ENROLMENT

5.1 IDENTIFYING PARTICIPANTS

Recruitment for the trial has been suspended until further notice on account of a USM requirement from the MHRA (12th February 2018).

[Procedure adopted prior to USM: Patients will be recruited from the gynaecological, outpatient clinics of participating centres (identified in both primary and secondary care), fitting around their current service provision. Figure 1 shows the different routes by which women may be identified and approached.**]**

5.1.1 Identification from GP databases

Recruitment for the trial has been suspended until further notice on account of a USM requirement from the MHRA (12th February 2018).

[Procedure adopted prior to USM: Patients with a history of HMB problems may be identified within GP practices. Databases will be screened using the HMB related Read codes, with access to these patient identification centres negotiated via the local Primary Care Network. The GP practices will send potentially eligible women a patient invitation letter, which will include a copy of the preliminary patient leaflet. The invitation letter will include the local contact details of the UCON research nurse, who can discuss the trial over the phone and if the patient is eligible make a hospital appointment. Once a hospital appointment is made, a letter confirming the appointment will be sent to the patient, which will include a patient information sheet.**]**

5.1.1.1 Raising awareness of the trial through community pharmacies

Recruitment for the trial has been suspended until further notice on account of a USM requirement from the MHRA (12th February 2018).

[Procedure adopted prior to USM: The Primary Care Network aim to reach out to as many patients as possible and this includes raising awareness of research through posters and leaflets in community pharmacy. Given patients with a history of HMB do not regularly present at their GP practice, UCON trial posters will be displayed in community pharmacy that have been selected by the Primacy Care Network. The poster will include brief information about the trial and provide local contact details of the UCON research nurse. Patients can discuss the trial over the phone and if the patient is eligible the UCON research nurse will make a hospital appointment. Once a hospital appointment is made, a letter confirming the appointment will be sent to the patient, which will include a patient information sheet.**]**

5.1.2 GP Referral to Secondary Care

Recruitment for the trial has been suspended until further notice on account of a USM requirement from the MHRA (12th February 2018).

[**Procedure adopted prior to USM**: Patients with existing HMB problems who present to a GP may then be referred to a participating hospital. GP practices in the catchment area of the participating centres will be made aware of the study and will be encouraged to discuss the trial with the woman, explaining a referral will be necessary. The UCON research nurse(s) will screen all patient referral letters to identify referred participants who have been introduced to the trial and arrange a gynaecology clinic appointment. Once a hospital appointment is made, a letter confirming the appointment will be sent to the patient, which will include a patient information sheet.]

5.1.3 Gynaecology Clinic Patient Identification

Recruitment for the trial has been suspended until further notice on account of a USM requirement from the MHRA (12th February 2018).

[**Procedure adopted prior to USM:** The UCON research nurses will screen the patient referral letters who have been referred by their GP to secondary care but who have not been introduced to the trial. Eligible patients will then receive a patient invitation letter along with a copy of the participant information sheet and be invited to contact the UCON research nurse to discuss the trial and/or make a hospital appointment. Once a hospital appointment is made, a letter confirming the appointment will be sent to the patient, which will include a patient information sheet.**]**

Please note, recruitment for the trial has been suspended until further notice on account of a USM requirement from the MHRA (12th February 2018.

Figure 1: Identification and screening of participants for the UCON trial

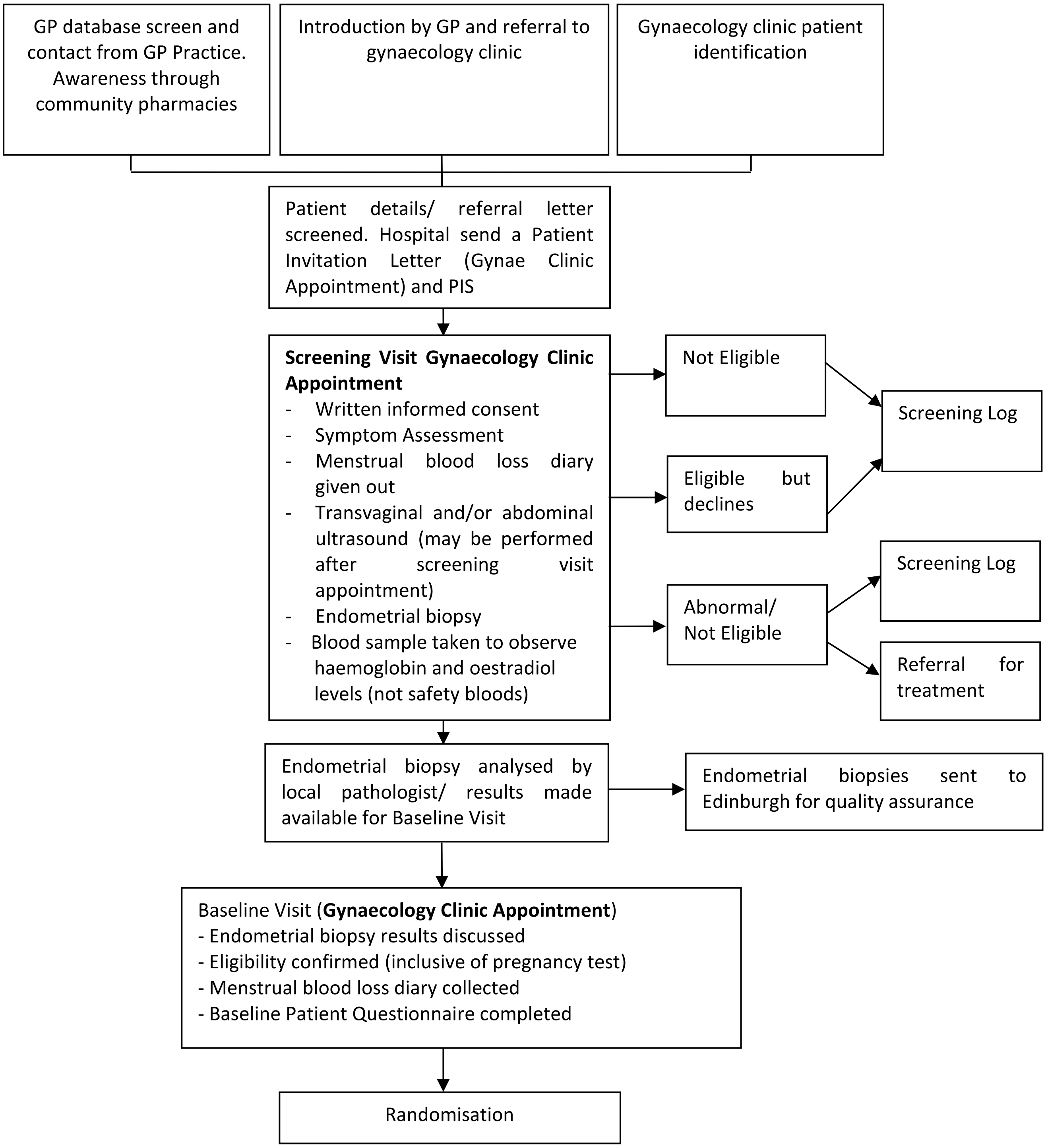

5.2 CONSENTING FOR SCREENING FOR ELIGIBILITY

Recruitment for the trial has been suspended until further notice on account of a USM requirement from the MHRA (12th February 2018).

[**Procedure adopted prior to USM:** All women who are referred to secondary care with HMB will be identified by the UCON research nurse(s) in each centre as a potential participant, prior to her outpatient appointment. The gynaecologist who will be providing her clinical care will discuss treatment options and establish eligibility based on history and preferences. The option to contribute to the mechanistic sub-study will instead be discussed in those centres able to contribute to the sub-study. Women who are confirmed pregnant are not eligible for the trial and participants would need to be prepared to avoid pregnancy for one year, so a discussion must be held about intentions to conceive.**]**

Consent to participate in UCON will be sought by the gynaecologist, and/ or by a research nurse. Women will be asked to consent to the UCON Trial in order that trial specific procedures, namely endometrial assessment using transabdominal and/ or transvaginal ultrasound and outpatient endometrial biopsy, can be undertaken. Women will be asked to confirm their consent by initialling the appropriate boxes on the consent form and signing in the presence of the person taking consent. Multiple copies will be available to ensure a copy is given to the women, one is kept in the patient notes, one in the local site file and one is sent to the UCON Trial Office.

Women who consent at this point should have the ultrasound(s) performed and biopsy taken at the same clinic appointment if at all possible. If a patient had an adequate ultrasound within the previous 3 months of the date of the baseline visit, and is documented in the clinical case records (medical notes), then the results may be used to determine eligibility within the UCON trial. The relevant member of the site team should complete an Ultrasound Form and send to the UCON Trial Office.

If a patient has confirmed submucosal fibroids >2cm at the screening ultrasound but is subsequently contraindicated by hysteroscopy (requested as part of standard clinical care pathway), this may confirm eligibility at the baseline visit.

All women should be given a menstrual blood loss diary containing the PBAC to take away and complete during their next period, which ideally will occur before the next gynaecology clinic appointment. The completed blood loss diary will need to be returned at the baseline visit prior to randomisation.

At the Royal Infirmary of Edinburgh the option of participating in addition in the mechanistic sub-study and undergoing additional endometrial biopsies and/ or MRI scans should be discussed. Women should be advised that these additional assessments are only applicable if they are randomly allocated to UPA.

All women approached should be recorded on the screening log(s), available in the investigator site file. This information will only be passed to the coordinating centre as an anonymous screening log.**]**

5.3 RE-CONSENTING PARTICIPANTS FOLLOWING THE USM

Participants actively taking UPA at the time of the USM will be permitted to complete their current 12 week course. In such cases, participants must be re-consented at their next visit (using updated participant information) to demonstrate that the updated safety information and additional blood tests have been discussed.

Alternatively participants may choose to stop UPA treatment – these participants do not need to re-consent .

5.4 CONFIRMATION OF ELIGIBILITY BEFORE RANDOMISATION/ ENTRY INTO THE MECHANISTIC SUB-STUDY

Recruitment for the trial has been suspended until further notice on account of a USM requirement from the MHRA (12th February 2018). Recruitment to this component of the study is already completed.

[**Procedure adopted prior to USM:** The participant should be invited to a baseline gynaecology clinic appointment by which time the results of the endometrial biopsy must be available. If a patient had an adequate endometrial biopsy within the previous 6 months of the date of the baseline visit, and is documented in the clinical case records (medical notes), then the results may be used to determine eligibility within the UCON trial. The relevant member of the site team should complete a Local Endometrial Biopsy Evaluation Form and send to the UCON Trial Office.

Normal findings from the local histopathology service will confirm eligibility for UCON, which should be relayed to the woman and continued consent established. At this point, the menstrual diary should be collected, if the woman has had a period in between appointments, and the other baseline questionnaires should be completed in clinic at this time.

Any pathological or suspicious findings from the biopsy should be investigated thoroughly and treated as appropriate outside of the UCON trial.]

5.5 INELIGIBLE AND NON-RECRUITED PARTICIPANTS

Participants who were consented prior to 12 February 2018 but were not randomised will have the reason for suspension of randomisation explained.

Such participants will return to routine care and an alternative treatment option for managing their complaint of HMB will be offered.

[**Procedure adopted prior to USM:** If a woman is screened but is not eligible for the trial due to a preference, contraindication or pathological reason for their HMB, or consent for randomisation is not given, an anonymous record of the case should be kept in the screening log. The screening log will include, age group, ethnic group, and the reason each patient not eligible for the trial. Women who consent and have an ultrasound and endometrial biopsy but are then found to be ineligible will be noted. The screening log should be kept in the site file and a copy sent to the UCON Trial Office, who will be able to identify women based on the information provided. This screening log information will inform updates to the funder regarding recruitment targets for UCON.]

5.6 RANDOMISATION

5.6.1 Randomisation Procedures

Randomisation for the trial has been suspended until further notice on account of a USM requirement from the MHRA (12th February 2018).

[**Procedure adopted prior to USM:** Immediately after eligibility has been established, baseline questionnaires have been completed, and once written informed consent has been obtained, the women may be randomised into the trial.

The Birmingham Clinical Trials Unit will provide third part web-based randomisation with telephone back-up. Patients are entered and randomised into the trial by logging into secure online randomisation available at https://www.trials.bham.ac.uk/UCON. Each centre and each randomiser will be provided with a unique log-in username and password in order to randomise a patient online. The online randomisation is available 24 hours a day, 7 days a week apart from short periods of scheduled maintenance and occasional network problems. Alternatively, investigators can make one Freephone telephone call (Tel - 0800 953 0274) to the randomisation service. Telephone randomisations are available Monday-Friday, 09:00-17:00.

Clinical Assessment and Randomisation Forms will be provided to investigators and may be used to collate the necessary information prior to randomisation. All eligibility related questions and data items on the Clinical Assessment and Randomisation Form will need to be answered before a trial number can be given. Only when all eligibility criteria and baseline data items have been provided will a trial number and treatment allocation be given followed by a confirmatory email sent to the randomising investigator, local Principal Investigator, local pharmacist and the research nurse.

A minimisation procedure using a computer based algorithm will be used to avoid chance imbalances in treatment allocation and the following potentially important variables:

Age: ≤35yrs or >35yrs
BMI: ≤25 kg/m^2 or >25 kg/m^2
Presence of any fibroid >2cm, as determined by the ultrasound scans
Duration of symptoms: < 1year or ≥1 year
Site: Individual Site
Agreement to enter sub-study: Both/ MRI only/ Biopsy Only/ Neither and N/A

In addition, to avoid any possibility of the treatment allocation becoming too predictable, we will include a random factor within the algorithm in which for a proportion of the allocations (1 in 5) true randomisation will be implemented rather than by using the minimised allocation.**]**

5.6.2 Treatment Allocation

Recruitment and randomisation for the trial has been suspended until further notice on account of a USM requirement from the MHRA (12th February 2018).

[**Procedure adopted prior to USM:** Participants will be randomised individually into the UCON trial in an equal ratio to either ulipristal acetate (UPA) or levonorgestrel releasing intrauterine system (LNG-IUS).**]**

Figure 2a: Patient pathway (Sites except Royal Infirmary of Edinburgh) Following USM

Screening Visit **(First Gynaecology Clinic Appointment)**
- Written informed consent
- Symptom Assessment
- Blood loss diary given out
- Ultrasound (standard clinical care ultrasound may be used as per Section 5.2)
- Endometrial biopsy (standard clinical care endometrial biopsy may be used as per Section 5.3)
- Blood sample taken

1 hour approx

Baseline Visit **(Gynae Clinic Appointment)**
- Endometrial biopsy results discussed
- Eligibility confirmed
- Blood loss dairy collected
- Baseline Patient Questionnaire completed

1 hour approx

Randomisation

Ulipristal

UPDATE: Complete current 12 week course of UPA or stop UPA (if continuing UPA, monthly liver function (blood) tests to be conducted).

No further courses to be initiated.

Liver Function test to be repeated 2-4 weeks after final UPA tablet

Complete a questionnaire/blood loss diary at:
3 months
6months 12 months
Post completed questionnaires to BCTU.

Biopsy should be obtained 4-12 weeks following cessation of UPA treatment (see section 5.6.5)

12 month Follow-up Appointment (Third Gynae Clinic Appointment)
- Ultrasound
- Blood sample

1 hour approx

Possible 15 month Endometrial biopsy if required
(Fourth Gynae Clinic Appointment)

20 mins approx

Possible 18 month Endometrial biopsy if required
(Fifth Gynae Clinic Appointment)

20 mins approx

Coil

Weeks 1 to 4 – Coil fitting appointment at hospital or GP

At 3 months complete a questionnaire/blood loss diary - post to BCTU

Complete 6 month postal questionnaire/blood loss diary - post to BCTU

Complete 12 month postal questionnaire/blood loss diary - post to BCTU

12 month Follow-up Appointment (Second or Third Gynae Clinic Appointment depending on when coil fitted)
- Ultrasound
- Blood sample

1 hour approx

Figure 3b: Patient pathway (Sites except Royal Infirmary of Edinburgh) Prior to USM

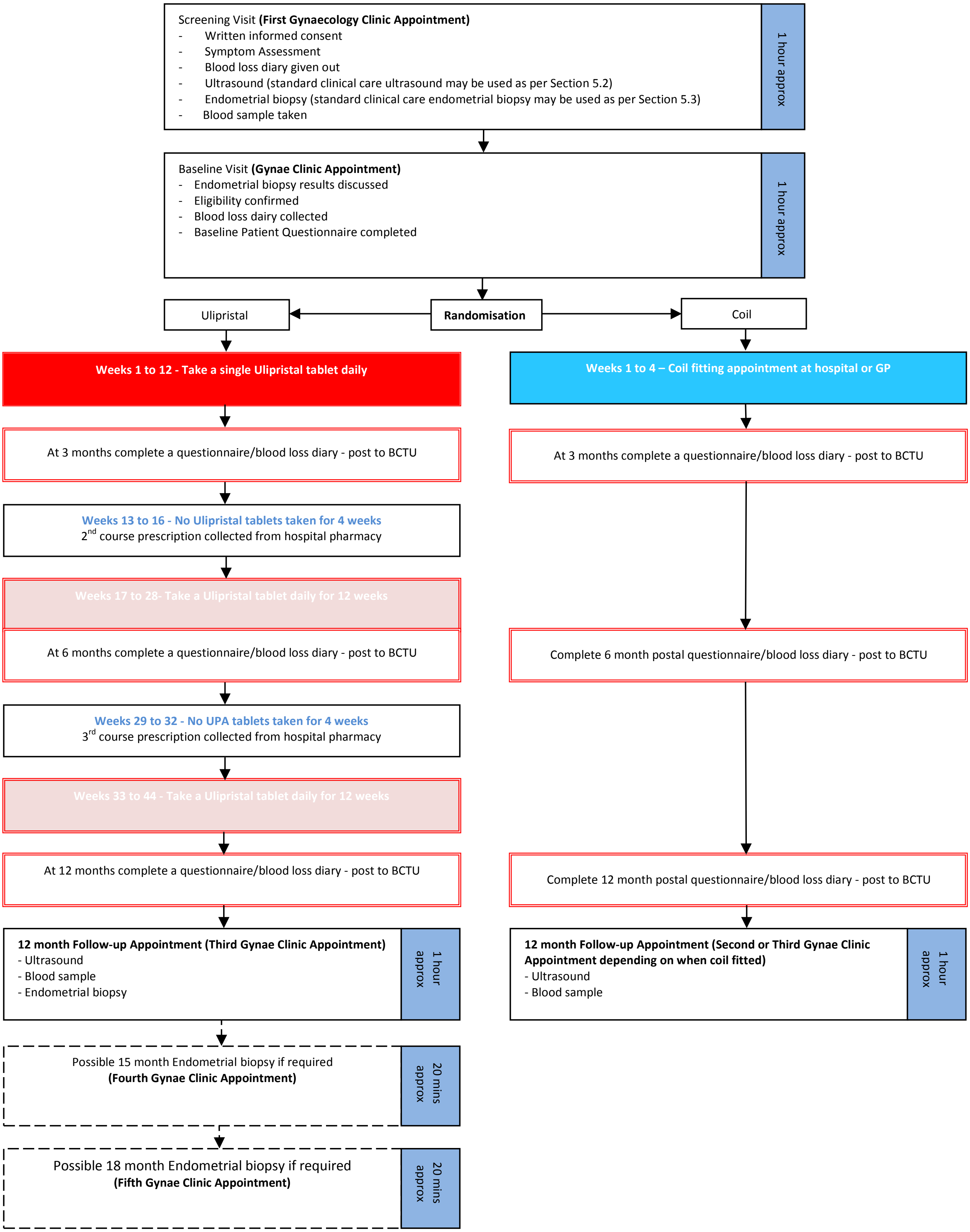

Figure 4: Patient pathway (Royal Infirmary of Edinburgh only)

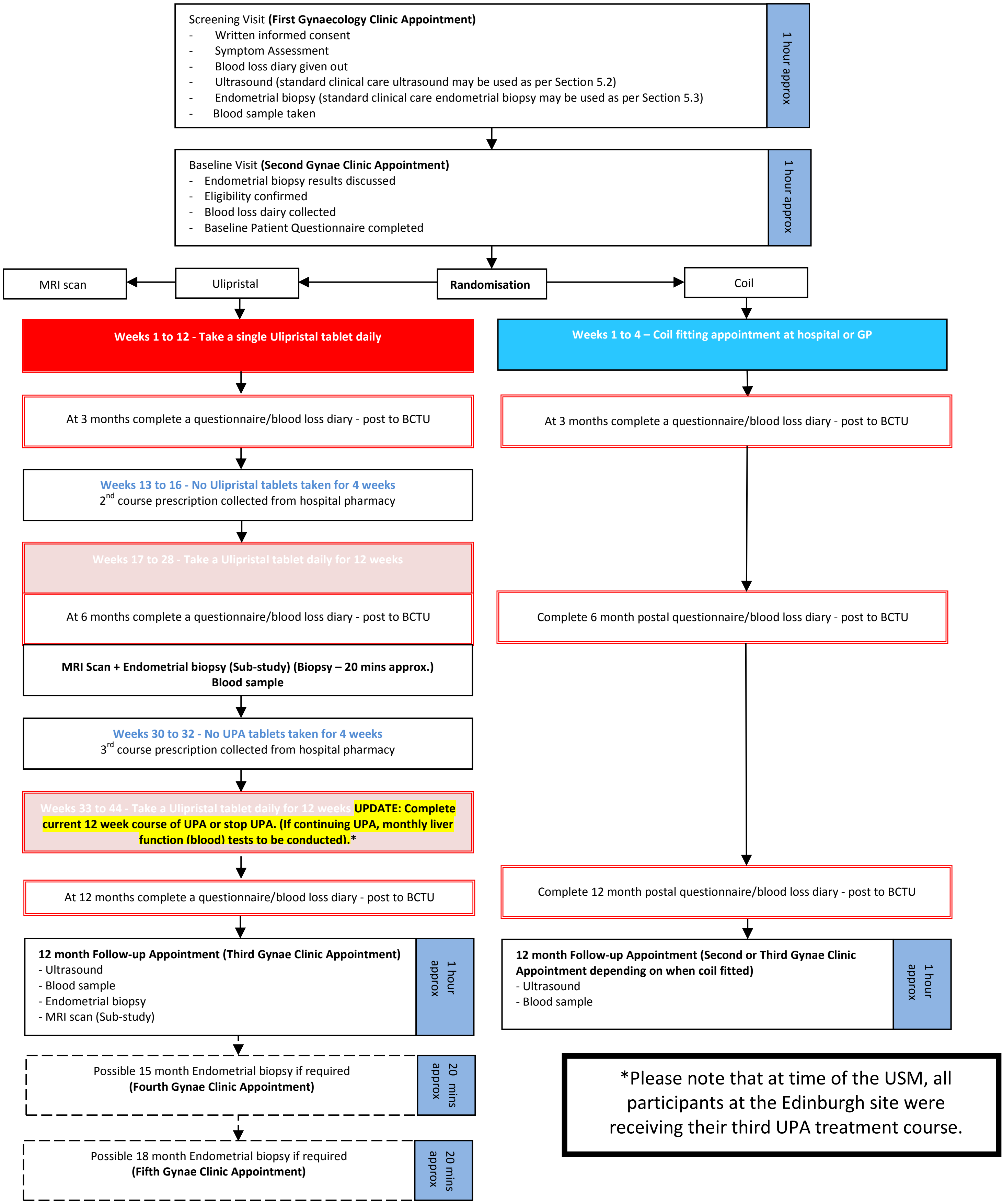

5.6.3 Baseline MRI for women in mechanistic sub-study (UPA)

Women opting for the mechanistic sub-study at the Royal Infirmary of Edinburgh will, have additional assessments that relate to this study only, but will otherwise complete the same study outcome as those randomised to the UPA group and be followed up in exactly the same manner. As part of the additional assessments will, where possible undergo an MRI scan during the secretory phase (second half of her cycle) of her menstrual cycle before commencing UPA (see Figure 3).

5.6.4 Blinding and Emergency Unblinding Procedures

As the treatments are so different in route of administration, the participants, investigators, research nurses and other attending clinicians cannot be blinded to the treatment allocation.

5.6.5 Stopping of treatment or Withdrawal of Study Participants

Trial treatment should continue until a woman has reached the 12 month assessment point unless:

- If randomised to UPA – current 12 week cycle of UPA may be completed (at participant's discretion), but no new UPA treatment cycle will be started (following MHRA drug alert February 2018).
- UPA treatment must be stopped if **transaminase levels are more than two times the upper limit of normal.**
- A known serious adverse reaction to UPA occurs and in the opinion of the investigator or clinically that it is medically necessary to withdraw the woman from trial treatment.
- A suspected unexpected serious adverse reaction occurs
- A participant changes mind and wishes to become pregnant
- The participant refuses to take the trial drug
- Women allocated LNG-IUS can retain the coil *in situ* for up to 5 years if they wish (depending on the product and manufacturer), and can have the coil replaced after this

With premature cessation of trial treatment, the trial staff will make every responsible effort to obtain, and record, information about the reasons for discontinuation, any adverse events and to follow-up the women for all safety and efficacy outcomes, as appropriate. An end of study endometrial biopsy for women discontinuing UPA treatment should be arranged after four weeks off treatment and no later than 12 weeks from cessation of treatment.

A participant may voluntarily withdraw participation in this study at any time. If a participant does not return for a scheduled visit or return a postal questionnaire, responsible attempts will be made to contact her and where possible, complete the patient reported outcome measures, and review compliance and adverse events. If a woman decides, after randomisation, she does not wish to take UPA, or wishes to have the LNG-IUS removed or wishes to withdraw from the trial for any reason (e.g. wishes to conceive or no longer interested in the research) then the woman should be strongly advised to have an end of study endometrial biopsy taken, at least four weeks after cessation of UPA. All participants wishing to withdraw from the trial will have the option of withdrawing from all aspects of the trial but may allow for continued use of data collected up to that point.

Where possible, the site and study team will aim to document the reason for withdrawing from the trial. Clear distinction will be made in the withdrawal/change of status form as to whether a participant is withdrawing from trial treatments whilst allowing further follow-up, or whether the participant refuses any follow-up. If a participant explicitly withdraws consent to have any further data recorded their decision will be respected and recorded. All communication surrounding the withdrawal or change of status will be noted in the patient's hospital records and in the trial database and no further data will be collected for that participant. For patients changing treatment but happy to allow further follow-up, the end of study ultrasound and bloods should be collected at the original intended time-point (weeks 49-57).

The target sample size of 220 patients includes a 20% loss to follow-up rate. Rates will be monitored to detect differential drop-out, which can bias clinical trial results and reduce the power of the trial to detect important differences.

5.6.1 Change of Treatment following USM

Following the USM, new cycles of UPA will not be initiated. In such instances, participants will be offered alternative options for treatment of their HMB. Stopping treatment or change to treatment must be documented on the UCON trial database using the Withdrawal/Change of Status Form (NOTE: the section regarding "Withdrawal from Trial" does not need to be completed if the woman is continuing in the trial). Change of treatment will also be collected as part of the questionnaires at standard assessment times.

6 INVESTIGATIONAL MEDICINAL PRODUCT(S)

6.1 STUDY DRUG

6.1.1 Study Drug Identification

The investigation medicinal products (IMPs) are ulipristal acetate and levonorgestrel-releasing intrauterine system will be used as a reference in its authorised form.

6.1.2 Ulipristal acetate (Comparator)

UPA is provided as a 5mg tablet. The trade name for UPA in the European Union is Esmya™ for treatment of uterine fibroids, and is marketed by Gedeon Richter.

6.1.3 Levonorgestrel (Reference)

The LNG-IUS is a contraceptive device that slowly releases a daily dose of 20 μg levonorgestrel into the uterine endometrium. It is a long acting reversible contraceptive preparation that requires removal and reinsertion every five years. LNG-IUS is approved for use as a contraceptive and for HMB and in the context of the current trial is manufactured by two companies. Bayer Pharma AG market their LNG-IUS under the name of Mirena™ and Actavis UK Ltd under the name of Levosert.

6.1.4 Study Drug Manufacturer and Supply

Each centre pharmacy will arrange an initial and continuing supply of ulipristal acetate and LNG-IUS through normal procurement procedures.

6.1.5 Marketing Authorisation Holder

The marketing authorisation holder for UPA (Esmya™) is Gedeon Richter (Hungary) Plc and the marketing authorisation number(s) is EU/1/12/750/001. The ATC code is G03XB02.

The marketing authorisation holder for LNG-IUS (Mirena™) is Bayer Plc and the marketing authorisation number(s) is PL00010/0547. The ATC code is G02BA03.

The marketing authorisation holder for LNG-IUS (Levosert) is Actavis UK Ltd and the marketing authorisation number(s) is PL30306/0438. The ATC code is G02BA03.

6.1.6 Labelling and Packaging

All details of trial drug supply; labelling, storage and preparation are as per the requirements of the Medicines for Human Use (Clinical Trials) Regulations 2004 and are detailed in the UCON Pharmacy Manual which is supplied to pharmacy at the time of site approval.

UPA dispensed by the pharmacy will require a trial specific label, complying with the Annex 13 of the EU Directive on Clinical Trials, 2004. Supplies of labels will be provided to the pharmacy at each participating hospital.

As LNG-IUS is not dispensed and is fitted according to the manufacturer's recommendations, so it will therefore not require trial specific labelling.

6.1.6 Storage

The UPA tablets must be kept in the blisters in the outer carton in order to protect from light. The blister packs are Alu-PVC/PE/PVDC blister and a pack may contain 28 or 84 tablets. There are no recommendations regarding temperature control of UPA, and so no specific temperature monitoring measures are required for the UCON Trial. The shelf life of UPA is 3 years.

Storage considerations are not applicable for LNG-IUS.

6.1.7 Dispensing and accountability

At randomisation, a packet of tablets will be dispensed to the woman. The pharmacist will receive notification of the name and trial number of the randomised woman and will prepare the trial treatment tablets for labelling and dispensing. The packet of UPA will contain 12 weeks' supply for use by one participant. An accountability log will be provided to record the dispensing of trial treatment.

6.1.8 Summary of Product Characteristics

The SmPC used for the reference safety information is located in the 'SmPC Reference Document' which is stored in the site file and pharmacy file.

SmPCs for each IMP can be accessed via the electronic Medicines Compendium (eMC) which contains up to date, easily accessible information about medicines licensed for use in the UK. The SmPC for each IMP and any updates during the recruitment and follow-up phase of the trial should be stored in the site file and pharmacy file.

6.2 DOSE AND DELIVERY OF IMPS

6.2.1 UPA

Randomisation was suspended on 12 February 2018 on account of a USM requirement from the MHRA (12th February 2018).

Participants already randomised to receive UPA may complete their current 12 week course No further courses will be initiated.

[**Procedure adopted prior to USM:** Those allocated to UPA will receive proprietary ulipristal acetate 5mg, orally, once daily.

A single tablet must be taken orally once daily with or without food, at approximately/ or as close as possible to the same time each day. The participant should start taking UPA within the first five days of starting their menstrual bleeding.

If a participant misses a dose, she should take UPA as soon as possible. If the dose was missed by more than 12 hours, the participant should not take the missed dose and simply resume the usual dosing schedule.

Women will be instructed to take UPA in 3 courses according to the following cyclical regime (± 5 days):

1. One 5mg tablet of UPA to be taken daily for 12 weeks then stopped for 4 weeks, when light vaginal bleeding may occur (withdrawal bleed).
2. After 4 weeks off treatment, regardless of whether they experience a withdrawal bleed, they should recommence UPA 5mg daily for another 12 weeks, then stop for 4 weeks, when they will expect to have a withdrawal bleed.
3. Repeat as for treatment courses (2).

We have chosen this regime as women and their clinicians will likely prefer a regime that has only one menstrual bleed between treatment courses and thus our study will be able to provide valuable data on this aspect of UPA treatment. **]**

6.2.2 LNG-IUS

Randomisation was suspended on 12 February 2018 on account of a USM requirement from the MHRA (12th February 2018).

The fitting of the LNG-IUS should be performed by the gynaecologist during outpatient visit, or later by a GP or at a sexual/ reproductive health clinic. If LNG-IUS is fitted within seven days of the onset of menstruation or withdrawal bleeding it will provide immediate contraceptive cover, otherwise barrier methods must be used for 14 days. The LNG-IUS can remain *in situ* up to for 5 years (depending on product and its respective shelf live) and should be removed by a competent practitioner, with immediate replacement if desired.

6.3 DOSE CHANGES

Participants already randomised to receive UPA may complete their current 12 week course No further courses will be initiated.

[**Procedure adopted prior to USM:** Each women randomised to UPA will take UPA 5mg, orally, once daily in 3 courses according to the cyclical regime stated in section 6.2.1:

The participant should start taking UPA within the first five days of starting their menstrual bleeding. or between 4 week and weeks 5 days from coming off treatment.**]**

6.4 PARTICIPANT COMPLIANCE

6.4.1 Maximising adherence of women to their allocated treatment.

We will try to avoid women not commencing the allocated treatment firstly by careful counselling with respect to childbearing intentions. Randomised women will either be provided with their UPA prescription immediately, or we will encourage women to have the LNG-IUS fitted promptly by the gynaecologist at the baseline clinic visit. To maintain adherence, women in the LNG-IUS group will be counselled to expect some disturbance to their menstrual cycle, but encouraged to persist.

6.4.2 Monitoring compliance

Follow-up questionnaires will ask for self-reported compliance to the allocated treatment. We may also collect data on adherence via a text message at various time points. LNG-IUS drug compliance will be participants who have had the coil fitted within 5 weeks post-randomisation.

UPA compliance will be those participants who report in the follow-up questionnaire to have taken the drug for at least 5 days per week. UPA drug compliance may also be evaluated by ‘pill-counting’.

6.5 SPECIAL WARNINGS AND PRECAUTIONS FOR USE FOR ULIPRISTAL ACETATE (UPA)

Five reports of serious liver injury, including four cases of hepatic failure needing liver transplantation, have been reported worldwide in women using UPA for uterine fibroids. UPA is currently under review by the European Medicines Agency and as such, precautionary measures have been implemented by the UCON Sponsor and trial to monitor for any potential risk:

- New treatment courses of UPA will not be initiated (including in women who have completed one or more treatment courses previously)
- Monthly liver function tests will be performed in women currently taking UPA

- UPA treatment will be stopped in any woman who develops transaminase levels more than 2 times the upper limit of normal. In such cases, participants will be closely monitored and referred for specialist hepatology evaluation if clinically indicated.
- Liver function tests will be repeated in all women 2 to 4 weeks after stopping UPA treatment.
- Transaminase levels will be checked in current or recent users of UPA (i.e. those who have taken UPA at least 30 days prior to 12 February 2018) who present with signs or symptoms suggestive of liver injury (such as nausea, vomiting, malaise, right hypochondrial pain, anorexia, asthenia, jaundice). If transaminase levels are more than 2 times the upper limit of normal. Such participants will be closely monitored and referred for specialist hepatology evaluation as clinically indicated.

All LFT results should be recorded on the trial database. Any abnormal LFT results identified should be recorded and if reaching seriousness criteria (as defined in section 10.1), should be reported as an SAE/SAR (as per section 10).

There is the potential for other medicinal products to affect ulipristal acetate and conversely the potential for ulipristal acetate to affect other medicinal products. For further details regarding guidance on prohibited and permitted medications please see sections 6.9.1and 6.9.2. For guidance regarding the potential interactions with other medications please see section 6.5.3.

6.5.1 Overdose

Experience with ulipristal acetate overdose is limited. Single doses up to 200 mg and daily doses of 50 mg for 10 consecutive days were administered to a limited number of subjects, and no severe or serious adverse reactions were reported.

6.5.2 Contraception

Concomitant use of UPA with progestagen-only pills, a progestagen-releasing intrauterine device or combined oral contraceptive pills is not recommended. Although a majority of women taking a therapeutic dose of ulipristal acetate are anovulatory, where contraception is required, the woman will be asked to use a barrier method with spermicidal foam/gel/film/cream/suppository, in line with MHRA contraception guidelines.

6.5.3 Potential drug interactions

Ulipristal acetate is not recommended for patients receiving P-glycoprotein (P-gp) substrates (e.g. dabigatran etexilate, digoxin).

Co-administration of moderate or potent Cytochrome P450 (CYP3A4) inhibitors (e.g. erythromycin propionate, ketoconazole, ritonavir, nefazodone) may lead to significant changes in plasma levels of ulipristal acetate and so women requiring potent drugs are not eligible for UCON and the use of UPA in those requiring moderate potency CYP should be review carefully. Concomitant use of mild CYP3A4 inhibitors is acceptable and no dose adjustment of UPA is considered necessary.

Patients receiving concomitant Cytochrome P450 (CYP3A4) inducers may have reduced plasma levels of UPA and so concomitant use potent CYP3A4 inducer, such as anti-convulsants (e.g.

carbamazepine, phenytoin) or anti-infectives (e.g. rifampicin, nevirapine) or St John's Wort is not recommended.

6.6 KNOWN ADVERSE REACTIONS FOR ULIPRISTAL ACETATE

A full list of known adverse reactions for UPA is given in SmPC Reference Document, whilst specific issues of concern are detailed here.

6.6.1 Endometrial changes

In 10-15% of women, thickening (> 16 mm by ultrasound or MRI at end of treatment) of the endometrium may occur. In addition, changes in the histology of the endometrium (PAECs) may be observed, that are different to endometrial hyperplasia. These changes are reversible after treatment cessation. More evidence regarding PAEC is discussed in Section 1.2.

6.6.2 Bleeding pattern

Participants should be informed that treatment with ulipristal acetate usually leads to a significant reduction in menstrual blood loss or amenorrhea within the first 10 days of treatment. Should the excessive bleeding persist, participants should notify their GP. Menstrual periods will generally return within 4 weeks after the end of the treatment course.

6.6.3 Other hormonal adverse events

Hot flushes were reported by 12.7% patients on average but the rates varied across trials. In PEARL II, the rates were 24% (10.5% moderate or severe) for UPA and 60.4% (39.6% moderate or severe) for leuprorelin-treated patients. In PEARL I, the rate of hot flushes was 1.0% for UPA and 0% for placebo.

Functional ovarian cysts were observed during and after treatment in 1.5% of patients and in most of the cases spontaneously disappeared within a few weeks.

6.6.1 Liver Injury (unconfirmed adverse reaction currently under investigation)

Five reports of serious liver injury, including four cases of hepatic failure needing liver transplantation, have been reported worldwide in women using UPA for uterine fibroids.

Women with severe hepatic impairment are excluded from the trial. Participants receiving UPA will undergo monthly liver function tests to monitor for signs of liver injury (see 6.5 for further details).

6.7 SPECIAL WARNINGS AND PRECAUTIONS FOR USE FOR LEVONORGESTREL

6.7.1 Fitting of the LNG-IUS

LNG-IUS fitting should ideally be performed by an experienced gynaecologist at the second clinic appointment. If this is not possible, a prescription can be given to enable the woman to go to a

sexual health clinic or her GP, for the coil to be fitted by a clinician as per standard clinical care and current service provision.

Perforation of the uterine corpus or cervix may occur, most commonly during insertion. This may be associated with severe pain and continued bleeding. If perforation is suspected the system should be removed as soon as possible and reported as a trial treatment withdrawal to the UCON Trial Office.

The insertion tube for LNG-IUS has been designed to minimise the risk of infections. Women should be told to be aware of symptoms and signs suggestive of pelvic infection and to go to her GP if at all concerned.

The RCOG guidelines suggest a women should be re-examined six weeks after insertion and further examinations should be performed where clinically indicated, but this will be left to the discretion of the gynaecologist to advise.

6.7.2 Potential drug interaction

The metabolism of progestogens may be increased by concomitant use of CYP3A4 inducers but the influence on these drugs on the contraceptive efficacy of the LNG-IUS has not been studied. However, it is not believed that CYP3A4 inducers will have a major importance, due to local mechanism of action of LNG-IUS.

6.8 KNOWN ADVERSE REACTIONS TO LEVONORGESTREL

Undesirable effects are more common during the first months after the insertion, and subside during prolonged use. A full list of known adverse reactions for LNG-IUS is given in Table whilst specific issues of concern are detailed here.

6.8.1 Bleeding irregularities

LNG-IUS usually achieves a significant reduction in menstrual blood loss in 3 to 6 months of treatment. Irregular bleeding/spotting may occur during the first months of therapy in pre-menopausal women. Some women's periods may even stop completely. Increased menstrual flow or unexpected bleeding may be indicative of expulsion.

6.8.2 Possibility of pregnancy

The LNG-IUS, when inserted properly, is an extremely effective contraceptive. The possibility of pregnancy should be considered in amenorrhoeic women if there are other symptoms, and expulsion should be excluded.

The absolute risk of ectopic pregnancy in LNG-IUS users is low. However, when a woman becomes pregnant with the LNG-IUS in situ, the relative likelihood of ectopic pregnancy is increased. The possibility of ectopic pregnancy should be considered in the case of lower abdominal pain - especially in connection with missed periods or if an amenorrhoeic woman starts bleeding.

6.9 OTHER MEDICATIONS

6.9.1 Permitted Medications

Drugs not listed as prohibited or have potential to interact (see Sections 6.5.3 and 6.7.2) are allowed.

6.9.2 Prohibited Medications

In order not to confound the effects of the trial treatments, the following should not be prescribed or taken by any participants whilst on trial treatment. Should the women withdraw or be excluded from the trial treatment but continue to provide data, continued regular use of below drugs should be noted. For patients who are 'coming off' some medications pre-randomisation then please refer to the wash out times specified in section 6.9.3.

- Mefenamic acid
- Tranexamic acid
- GnRH analogues
- Progestagen-only contraceptive
- Any progestagen-releasing intrauterine device (except if allocated within UCON)
- Any combined oral contraceptive pills

6.9.3 Wash out times

• GnRH agonist/ antagonist: 3 to 6 month sustained-release preparation	52 wks
• Immediate or monthly sustained-release GnRH agonist preparation	26 wks
• Sex steroid: Progestins (systemic/ progestin-releasing intra-uterine system)	4 wks
• Oral contraceptive	4 wks
• Mefenamic acid or antifibrinolytic drugs such as tranexamic acid	1 wk

7 STUDY ASSESSMENTS

Due to the different nature of the IMPs, the timing and format of the study assessments will differ slightly between the groups.

7.1 STUDY ASSESSMENTS OVERVIEW

A summary of actions and assessments undertaken, and data collected, at each time point is shown in **the Trial Schema (**

Appendix 1: TRIAL SCHEMA

Appendix 1**)**. Women who have consented and randomised to the mechanistic sub-study will have additional assessments that relate to this study only, but will otherwise complete the same study outcome as those randomised to the UPA group and be followed up in exactly the same manner.

7.2 TIMING OF STUDY ASSESSMENTS

The overriding principles for the timing of the follow-up study assessments are:

1. The patient completed questionnaires should ideally be completed in the final week of each on-treatment cycle or equivalent for the UPA group, and at an equivalent time for the LNG-IUS group. Participants who stop treatment should complete questionnaires as originally scheduled (i.e. approximately 3, 6 and 12 months post randomisation).

2. The menstrual blood loss diary whilst on treatment should ideally be completed over the final four weeks of each treatment cycle in the UPA group, and at an equivalent time for the LNG-IUS group. The UPA group will also be asked to complete the diary during the first 4 weeks off treatment before the start of the next treatment cycle (as per the cyclical regime stated in section 6.2.1).

3. The post-treatment endometrial biopsy should be completed after 4 weeks off treatment, which would be approximately 48 weeks after UPA was commenced.

4. Should PAECs be observed in the post-treatment biopsy specimen in the UPA group, a repeat endometrial biopsy should be taken around 13 weeks after the completion of treatment, and then again around 26 weeks post-treatment if PAECs persist.

5. The endometrial biopsy, blood sample and MRI at time point 26 weeks in the sub-study should be in the final week of the second UPA cycle, to determine the features of the endometrium while receiving treatment.

A timeline for the treatment regimens and completion of the assessments is shown in Figure 4.

Figure 5: Timeline for the treatment regime for UPA and LNG-IUS, and study assessments (Prior to USM)

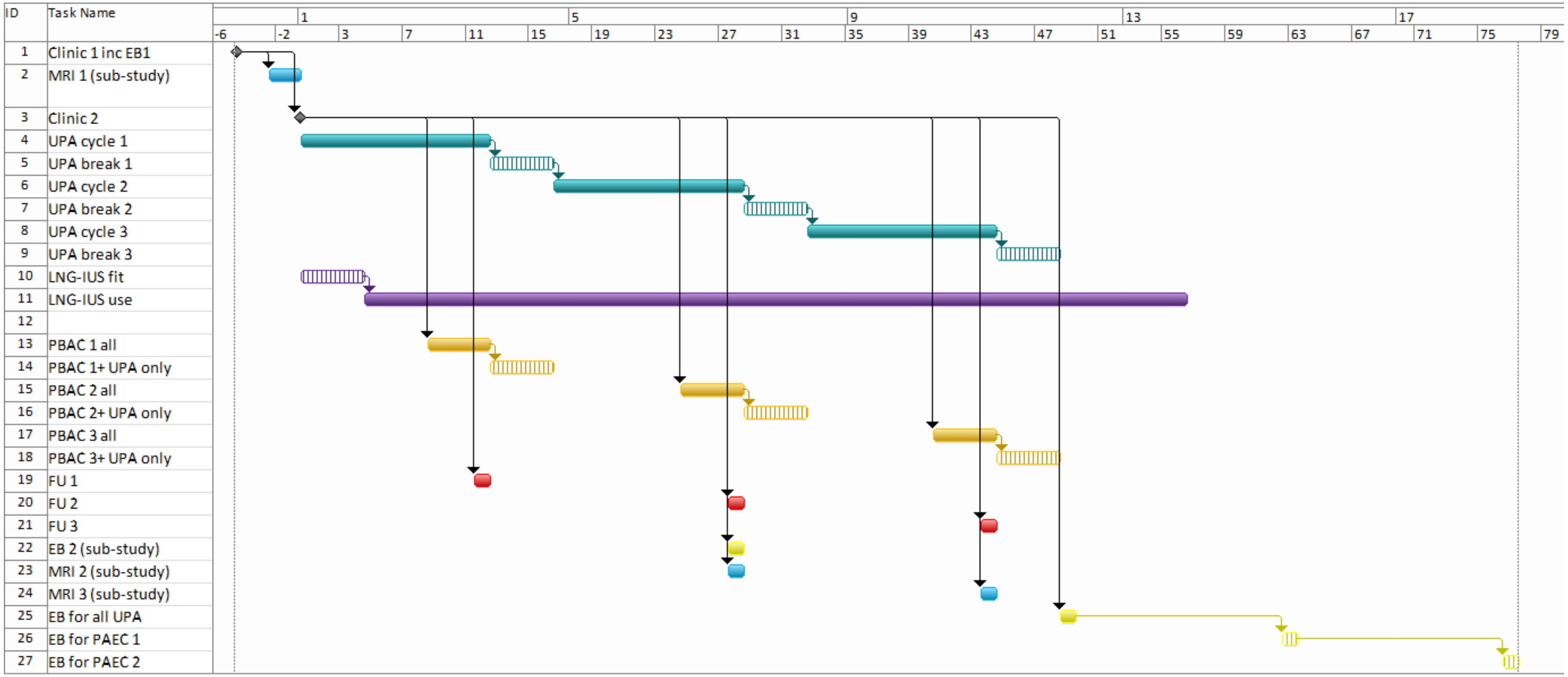

Key: EB endometrial biopsy; FU patient questionnaire

7.3 OUTCOMES COLLECTED AT STUDY ASSESSMENTS

Table 1 shows the outcomes collected at each time point in the UCON trial and mechanistic sub-study.

Table 1: Schedule of outcome assessment for UCON trial and mechanistic studies

(X) optional ((X)) dependent upon endometrial assessment at previous time point

Timepoint	Screening (1)	Baseline (2)	3 months (approx) (3)	6 months (approx) (4)	12 months (approx) (5)	Post-treatment 1 (6)	Post-treatment 2 (7)
Written informed consent	X						
Patient questionnaires (MMAS, UFS-QOL, EQ-5D-5L, ICE-CAP, SAQ)		X	X	X	X		
Other patient reported outcomes (compliance, adverse events, willingness to recommend to a friend, rating of treatment, satisfaction of treatment)					X		
Menstrual bleeding diary	X		X	X	X		
Blood sample, to observe haemoglobin and oestradiol levels (not safety bloods)	X				X		
Ultrasound pelvic assessment	X				X		
Endometrial biopsy	X				X UPA only **		
Endometrial biopsy – additional for women in UPA group who exhibit PAEC						((X)) UPA only	((X)) UPA only
Follow up outpatient appointment to discuss post-trial treatment options						(X) UPA only	
Liver Function Test (if indicated)			X***				
**Purposive samples 1 and 2 (Edinburgh sub-study only)**							
Endometrial biopsy and blood test	X			X UPA only	X UPA only	((X)) UPA only	((X)) UPA only
MRI (DCE-MRI and high resolution structural MRI)		X		X UPA only	X UPA (+) only		

(X) optional ((X)) dependent upon endometrial assessment at previous time point

(+) MRI to be performed in final week of treatment

* Endometrial biopsies will be stored at the University of Edinburgh Female Reproductive Tract Tissue Resource (10/S1402/59) and 16/ES/0007.

** An end of study endometrial biopsy for women discontinuing UPA treatment should be arranged four weeks post cessation.

*** As per the Urgent Safety Measure: LFTs will be performed monthly during the 12 week treatment period for women currently taking UPA. A LFT must also be performed 2-4 weeks after the final dose of UPA. LFTs must include transaminases (ALT and AST). LFT results must be recorded on the UCON trial database.

8 DATA COLLECTION

8.1 DATA COLLECTION FORMS

Data for the purpose of assessing the efficacy and safety within the UCON trial will be collected from the women, her gynaecologist and the histopathologist on a number of data collection (case report) forms.

8.1.1 Participant Questionnaire

The participant questionnaire is a booklet containing a number of validated instruments and questions completely independently by the participant. The booklet at baseline will contain:

- Menorrhagia Multi-Attribute Scale (MMAS)
- Uterine Fibroid Symptom and Quality of Life (UFS-QoL)
- Quality of Life (EuroQoL *EQ-5D-5L)) and (ICECAP-A)*
- Sexual Activity Questionnaire (SAQ)Cycle regularity and duration
- Visual analogue scales (0-10) for pelvic pain during periods, intercourse and at other times

At the three follow-up timepoints, the following additional data will be sought:

- Adherence to UPA dosing schedule, as subjectively reported on an ordinal scale by the participant (for those allocated to UPA).
- Adherence to LNG-IUS for those allocated this group.
- Discontinuation or changes in allocated treatment.
- Any hospitalisations, or further investigations or treatments from a gynaecologist.
- Any pregnancies.
- Serious adverse events reported by participants, principally those that are unexpected or are known and relevant to the trial treatments.

The booklet will either be sent out in paper form by post, be emailed as a data-form enabled attachment, be via a password protected web form or be presented to the woman in an outpatient clinic. Various ways in which to contact women (land line and mobile telephone, email, address) will be collected and all may be used in the process of collecting the data.

8.1.2 Menstrual Diary

The standard pictorial blood assessment chart (PBAC) (14) will be given to the woman at the first clinic visit to allow her to complete during her next menstruation and return it at the second clinic visit, which should be scheduled to be at least 4 weeks after the first.

Both UPA and LNG-IUS may induce amenorrhea (absence of bleeding) in some women, so whilst on treatment, the concept of a regular cycle is problematic. A modified menstrual diary, with ordinal questions regarding bleeding each day, will be used to establish the degree of menstrual bleeding. This will be completed for 28 days from week 8 for women in the LNG-IUS group and for 56 days

from week 8 in the UPA group, then again at week 24 and week 40, for 28 and 56 days for the LNG-IUS and UPA groups, respectively.

8.1.3 Clinical Assessment and Randomisation Form

At the first clinic visit, the gynaecological clinical history of the woman will be taken and details of duration of HMB symptoms, previous gynaecological treatment for HMB, and the contraceptive use and needs of the woman will be collected alongside basic demographic details. A blood sample to observe serum haemoglobin and oestradiol levels will be taken and results recorded in the clinical assessment and randomisation form.

8.1.4 Ultrasound Form

A pelvic examination by transabdominal and/or transvaginal ultrasound will be undertaken and cardinal features noted as possible presence of fibroids and uterine size and size of largest fibroid to make the comparison between baseline and 12 month follow-up possible.

8.1.5 Clinical Assessment and Randomisation Form and Screening Logs

The Clinical assessment and Randomisation Form is a checklist for eligibility and key prognostic details needed for minimisation within the randomisation. The Clinical assessment and Randomisation Form is completed by the investigator or UCON research nurse before randomisation.

The Screening Logs, described in Section 5.5, will record basic details of all women approached, including those who are found to be ineligible and those that decline to participate. This should be kept up to date by the UCON research nurse.

8.1.6 Endometrial Biopsy Report Form

The local consultant histopathologist will report on the morphology and cellular architecture of the endometrial biopsy sample on a local report form. The baseline biopsy and the 48 week post treatment biopsy in the UPA group will be assessed using standard techniques to identify PAECs. Independent analysis of the slides taken from the endometrial samples by the Lead Pathologist at the University of Edinburgh will be recorded on a standardised report form.

8.1.7 End of Study Visit Form

The research nurse will contact each participant to arrange their end of study visit (follow-up assessment) which will ideally take place between approximately weeks 49 and 57. The assessment will include confirmation of whether there was a change to treatment during trial participation, satisfaction with treatment, participant rating of treatment and whether they would recommend to a friend and onward treatment intention.

Participants in the LNG-IUS group will undergo an ultrasound and have a blood sample taken. Participants in the UPA group will undergo an ultrasound, endometrial biopsy and have a blood sample taken.

For participants in the UPA mechanistic sub-study will undergo an ultrasound, endometrial biopsy, MRI scan and have a blood sample taken.

8.1.8 Serious Adverse Event Form

This will collect details of all SAEs are defined and description in Section 10.5.

8.1.9 Mechanistic Study Data Forms

Data forms pertinent to the assays and analyses being undertaken on the endometrial biopsy and MRI scans taken for the sub-study will be used to standardise data collected.

8.1.10 Liver Function Test Results

As per the Urgent Safety Measure: LFTs will be performed monthly during the 12 week treatment period for women currently taking UPA. A LFT must also be performed 2-4 weeks after the final dose of UPA. LFTs must include transaminases (ALT and AST). LFT results must be recorded on the UCON trial database.

8.2 SOURCE DATA

For the purposes of the UCON trial, source data comprises of:

- Clinical Assessment and Randomisation Form
- Patient questionnaire and menstrual diary
- Clinical notes
- Blood sample for haemoglobin and oestradiol analysis
- Ultrasound
- Endometrial biopsy sample for standard histopathological analysis.
- End of Study Visit Form

8.3 DATA MANAGEMENT

Data from the Clinical Assessment and Randomisation Form and Endometrial Biopsy Report Form and should be entered on to the secure online UCON database as soon as possible after collection by the research nurse, investigator or histopathologist, who will be allocated personal usernames and passwords that restrict access to participants at their centre. Alternatively, paper forms can be sent to the UCON Trial Office for central input. Patient completed forms will be returned directly to the UCON Trial Office for data entry.

Data validation is built into the online database, so that range, date and logic checks are performed at the point of data entry. Email, text message and letter reminders will be sent to the research nurses or participants for missing data forms, missing data or data inconsistencies.

8.4 QUALITY ASSURANCE OF ENDOMETRIAL BIOPSY ASSESSMENTS

The assessment of endometrial biopsies should be undertaken by a consultant histopathologist with expertise in endometrial analysis. For quality assurance, a second assessment will be undertaken by the lead pathologist for UCON, Professor Alistair Williams at the University of Edinburgh. The histopathologist or research nurse should send the screening endometrial biopsy sample slides to Professor Williams for second reading in secure shipping containers (available from the UCON Trial Office upon request).

Slides taken from the samples will be labelled with:

- Trial No.
- Patient Initials
- Slide Number/Accession Number
- Date biopsy taken

and sent for second reading in secure shipping containers which may be provided by the UCON Trial Office upon request.

For the baseline endometrial biopsy, the second assessment in Edinburgh will not be used to confirm eligibility for the trial and therefore slides may be sent in batches.

For the final post-treatment biopsy in the UPA group, a local assessment will be under taken and the slides sent promptly to Edinburgh. Whilst the local assessment will be noted, the second Edinburgh review of the slides, undertaken without knowledge of the local assessment, will determine the presence or absence of PAEC for the purpose of the trial. Those women with PAECs as confirmed by Edinburgh will be asked to return for endometrial biopsies at 13 weeks, and if necessary 26 weeks after completion of the final UPA course.

All slides will be returned to their originating hospitals after the second assessment.

8.5 MECHANISTIC SUB-STUDY

8.5.1 Endometrial tissue function

An overview of approach to analysis of tissue-specific impacts of UPA on endometrial tissue function are summarised in Table 2 below. The choice of target endpoints has been informed by studies in our own laboratory that have highlighted the impact(s) of progestins, progestin receptor antagonists and receptor modulators levonorgestrel, mifepristone, asoprisnil) on endometrial tissue function, *examples given but not limited to those end points identified in Table 2* (17-19).

Table 2: An overview of approach to analysis of tissue-specific impacts of UPA on endometrial tissue function

Process	Endpoint	Details of analytical method
Tissue morphology	H&E stain histology, number of mitoses and mitochondria	Analysis by expert pathologist<br>qRT-PCR mitochrondrial markers
Vascular morphology	Endothelial and perivascular cell function/morphology	Double staining for CD31 (endothelial cells) and smooth muscle actin<br>Masson Trichrome staining for collagen
Regulation of cell number	Cell proliferation, apoptosis and autophagy	Immunostaining for Ki67, PH3(proliferation) or cleaved caspase 3, microtubule-associated protein 1 light chain 3 alpha (death/survival), gamma H2AX (senescence)
Steroid hormone signalling	Cell-specific pattern of expression of steroid hormone receptors and steroid metabolizing enzymes	Single and double immunostaining for PR, ERalpha, ERbeta, AR, GR
Inflammation	Immune cell complement and inflammatory mediators	qRT-PCR IL15.<br>Immunostaining for CD56, CD68, neutrophil elastase, mast cell tryptase and pan leucocyte marker CD45
Pre-malignant change	Comparison with normal endometrium and endometrial hyperplasia	qRT-PCR, double fluorescent immunohistochemistry PTEN, E-Cadherin, Snail 1, vimentin, PAX2, telomerase (hTERT)

8.5.2 Uterine and fibroid function

The aim of the MRI sub-study is to investigate the hypothesis that UPA will reduce blood flow and blood volume in the endometrium and myometrium in women with HMB. In particular, dynamic contrast enhanced MRI (DCE-MRI) and high spatial resolution structural MRI will be obtained in a subgroup of 20 women treated with UPA. DCE-MRI, combined with pharmacokinetic modelling, yields quantitative estimates of physiological parameters, including tissue blood flow, blood volume fraction and endothelial permeability, as well as volume fraction of the extracellular extravascular space. Structural MRI provides high resolution images suitable for structural segmentation and radiological evaluation that, when combined with design based stereological analysis (20), yield accurate and precise measurements of uterine and fibroid volume for early assessment of treatment response.

Scanning will be performed at baseline, and at 6 and 12 months following commencement of treatment. Scans will take place during the secretory phase of the menstrual cycle at baseline and in the week prior to the end of the second and third cycles of UPA, weeks 27 and 43 respectively.

Structural images will be evaluated clinically by an experienced radiologist, and stereological analysis performed to determine the volumes of the uterus and of any fibroids. Modern design based stereological methods will measure total volume of endo- and myo-metrial compartments, and

volume, type and location of individual fibroids, with mathematically predicted precision, on high resolution MR images.

Dynamic Contrast Enhanced (DCE)-MRI will be used to measure uterine tissue perfusion. Contrast agent concentration is modelled as exchange between the blood plasma and extracellular interstitial spaces, providing maps of tissue blood flow, blood and interstitial volume fraction and artery to tissue delay (i.e. lag) time. DCE-MRI data will be analysed using the well-established adiabatic approximation to tissue homogeneity (AAHT) model, to generate pharmacokinetic maps of blood flow, blood volume and extracellular extravascular volume fraction; these will be used to extract representative values for endometrium, junctional zone, outer myometrium and fibroid tissue.

Both MRI approaches are novel in the context of SPRM administration, so will be piloted in the first patients, who may require repeat scans with a refined protocol.

9 STATISTICS AND DATA ANALYSIS

9.1 SAMPLE SIZE CALCULATION

9.1.1 RCT

The trial has been designed to be able to detect a clinically useful difference in MMAS score between the two groups at twelve months with high power. The ECLIPSE Trial, which evaluated the effectiveness of LNG-IUS against Standard treatment for HMB (21) using MMAS as the primary outcome, demonstrated a difference of 13 points between the groups with a standard deviation of 24 points. This difference considered to be clinically meaningful (22) and is equivalent to approximately 0.5 standard deviations. To detect a difference of this size with 90% power (p=0.05) would require 86 women in each group (172 in total). To allow for a 20% loss to follow-up or pregnancy, the sample size has been inflated to 220 women in total.

9.1.2 Mechanistic sub-study

For the mechanistic evaluation 20 samples would give >90% power (p=0.05) to detect a change from baseline in for example, PH3 immuno-score, in the endometrium assuming similar effect sizes (>1 standard deviation) as to those seen in previous studies of other PRMs (19).

9.1.3 Anticipated recruitment period

Recruitment will take place over a minimum of 24 months from at least 5 centres, with a target of 2 patients/centre/month. All centres have large HMB clinics, are experienced in recruiting to RCTs and the BCTU has a track record of completing RCTs in women's health.

9.2 PROPOSED ANALYSES - RCT

The analysis will be by intention to treat. Every attempt will be made to gather data on all women randomised, irrespective of compliance with the treatment protocol. Appropriate baseline characteristics, split by treatment group, will be presented for each outcome. Point estimates, 95% confidence intervals and p-values from two-sided tests will be reported. A full Statistical Analysis

Plan will be drafted prior to any analysis and provided to independent Data Monitoring and Ethics Committee for review.

9.2.1 Primary analysis

A linear regression model will be used to estimate differences in MMAS responses between the two groups at each time point. Baseline score and the minimisation variables (listed in section 5.5.1) will be included in the model as covariates. The statistical significance of the treatment group variable will be determined by an associated chi-squared test.

9.2.2 Secondary analysis

MMAS scores at three and six months follow-up will be analysed as per the primary analysis. Data from the other continuous measures (UFS-QOL, EQ-5D, ICE-CAP, VAS and SAQ) will be analysed in a similar fashion to the MMAS scores. Further exploratory analysis using a repeated measures model will also be used for these continuous measures to examine differences over all time-points. Bleeding diary scores will be converted into categories including the proportion with amenorrhoea (=0) and heavy bleeding (>=100). They will be analysed using relative risks and chi-squared tests. Time from randomisation to surgery (hysterectomy or endometrial ablation) will be analysed by log-rank test with a Cox Proportional Hazard (PH) model also built if the assumptions of proportionality are met. Other outcome measures (Likert responses, Likert ordinal responses, satisfaction) will be analysed using standard methods (tests for trend, absolute and relative risks). Paired t-tests will be used to examine differences within groups over time.

9.2.3 Sub-group analysis

Subgroup analyses will be limited to the same variables which were used as minimisation variables (see Section 5.6.1). Tests for statistical heterogeneity (e.g. by including treatment group by subgroup interaction parameter in the linear regression model) will be performed prior to any examination of effect estimate within subgroups. Our hypothesis is that UPA may be more effective in older (>35 years) and heavier (BMI>25) women and those who have a presence of fibroid or have experienced symptoms for longer than a year.

9.2.4 Handling missing data and other sensitivity analysis

Every attempt will be used to collect full follow-up data on all women. In particular, the trial team will endeavour to follow-up participants even after protocol treatment violation. It is thus anticipated that missing data will be minimal. Patients with missing primary outcome data will not be included in the primary analysis. This presents a risk of bias. Thus, secondary sensitivity analyses will be performed to investigate the impact of any missing data for the primary, as well as any important secondary outcome. This will include worst (for those randomly missing) and best case assumptions (for those not able to complete the primary outcome as they no longer can have menstrual bleeding, i.e. because they have had a hysterectomy). We will also simulate missing responses using a multiple imputation approach. To explore the sensitivity of the primary MMAS

analysis to any ceiling effects (i.e. a high proportion returning a maximum responses, i.e. no problems with bleeding), a Tobit regression model will also be implemented.

9.2.5 Timing of assessments

An interim report including the analysis of major endpoints will be provided in strict confidence to a Data Monitoring Committee at intervals of at least 12 months, or as to a timetable agreed by the DMC prior to study commencement (see Section 11.4 for further details on trial data monitoring including the use of pragmatic stopping criteria). Final analysis will be performed once all women have completed twelve months follow-up.

9.3 PROPOSED ANALYSES – MECHANISTIC STUDY

Outcomes for the n=20 prospectively studied women biopsied at 6 and 12 months will be compared to each participant's own baseline biopsy as a control. Outcomes (e.g. PH3 immunoscore changes within groups over time) will be analysing using paired t-tests following an appropriate transformation and also by using non-parametric methods (e.g. Wilcoxon signed rank test) for confirmation.

10 ADVERSE EVENTS AND PHARMACOVIGILANCE

The Investigator is responsible for the detection and documentation of events meeting the criteria and definitions detailed below. These tasks may also be delegated to a qualified member(s) of the research team. Assessment of events may be delegated to other suitably qualified physicians in the research team who are trained in recording and reporting adverse events.

Participants will be instructed to contact their investigator at any time after randomisation if any symptoms develop. In the case of an AE, the Investigator should initiate the appropriate treatment according to their medical judgment.

10.1 DEFINITIONS

An **adverse event** (AE) is any untoward medical occurrence in a clinical trial participant which does not necessarily have a causal relationship with an investigational medicinal product (IMP).

An **adverse reaction** (AR) is any untoward and unintended response to an IMP which is related to any dose administered to that participant.

A **serious adverse event** (SAE), **serious adverse reaction** (SAR). Any AE or AR that at any dose:

- results in death of the clinical trial participant;
- is life threatening*;

- requires in-patient hospitalisation^ or prolongation of existing hospitalisation;
- results in persistent or significant disability or incapacity;
- consists of a congenital anomaly or birth defect;
- results in any other significant medical event not meeting the criteria above.

*Life-threatening in the definition of an SAE or SAR refers to an event where the participant was at risk of death at the time of the event. It does not refer to an event which hypothetically might have caused death if it were more severe.

^Any hospitalisation that was planned prior to randomisation will not meet SAE criteria.

A suspected unexpected serious adverse reaction (SUSAR) is any AR that is classified as serious and is suspected to be caused by either IMP, that it is not consistent with the information about the IMP in the Summary of Product Characteristics (SmPC).

10.2 IDENTIFYING AES AND SAES

There may be expected and unexpected adverse reactions, which may be minor or serious, associated with UPA and LNG-IUS when used in women affected by HMB. The adverse event profile for LNG-IUS is well defined, as the system has been licenced for over a decade, and hence the collection of expected adverse events is not required. For example, elective admission for LNG-IUS insertion or elective admission for hysterectomy would not need to be classified as a serious adverse event. The focus for safety reporting of UPA is on changes to the endometrium.

Open-ended and non-leading verbal questioning of the participant will be used to enquire about AE/SAE occurrence during each study visit and in postal questionnaires. Participants will also be asked if they have been admitted to hospital, had any gynaecological treatments, used any new medicines or changed concomitant medication regimens. If there is any doubt as to whether a clinical observation is an SAE, the event will be recorded and reported.

AEs and SAEs may also be identified via information from the endometrial biopsy, which will be recorded on the histopathology report form and SAE form if necessary. Untoward findings may incidentally be found in women in the mechanistic study, for example unexpected pelvic masses seen on by MR imaging. These will be reported to the local Principal Investigator who will assess whether they constitute an AE or SAE and the appropriate clinical management will be determined. If any untoward findings meet seriousness criteria they will be subject to onward reporting.

All diagnoses of endometrial cancer, ovarian cancer, cervical cancer, breast cancer or ductal carcinoma must be reported as a SAE.

10.3 RECORDING AES AND SAES

When an AE/SAE occurs, it is the responsibility of the Investigator to review all documentation (e.g. hospital notes, laboratory and diagnostic reports) related to the event. The investigator will then record all relevant information regarding SAEs on the SAE form (if the AE meets the criteria of serious) and record all unexpected AEs in both groups on the AE log. Expected AEs do not need to be recorded, unless they meet seriousness criteria, listed in the SmPC Reference Document.

10.4 ASSESSMENT OF AES AND SAES

Seriousness, causality, severity and expectedness will be assessed by the Principal Investigator or another suitably qualified physician in the research team. Cases that are considered serious, possibly, probably or definitely related to either IMP and unexpected (i.e. SUSARs) will be thoroughly investigated.

The Investigator is responsible for assessing each AE. This may be delegated to other suitably qualified physicians in the research team who are trained in recording AEs and recording and reporting SAEs.

The Chief Investigator (CI) may not downgrade an event that has been assessed by an Investigator as an SAE or SUSAR, but can upgrade an AE to an SAE, SAR or SUSAR if appropriate.

10.4.1 Assessment of Seriousness

The Investigator will make an assessment of seriousness as defined in Section 10.1.

10.4.2 Assessment of Causality

The Investigator will make an assessment of whether the AE/SAE is likely to be related to the IMP according to the definitions below.

- <u>Unrelated</u>: where an event is not considered to be related to the IMP.
- <u>Possibly Related:</u> The nature of the event, the underlying medical condition, concomitant medication or temporal relationship make it possible that the AE has a causal relationship to the study drug. The assessment of causality will be made against the reference safety information found in the Section 4.8 of the relevant Summary of Product Characteristics.

The reference safety information i.e. known undesirable effects are detailed in the SmPC Reference Document which is stored in the site file and pharmacy file (see also Section 6.1.8).

Where there are concomitant medications, if the AE is considered to be related to an interaction between the IMP and the other medication, or where the AE might be linked to either the IMP or the other medication but cannot be clearly attributed to either one of these, the event will be

considered as an AR. Alternative causes such as nature of the HMB, other risk factors and the temporal relationship of the event to the treatment should be considered and investigated.

10.4.3 Assessment of Expectedness

If an event is judged to be an AR, the evaluation of expectedness will be made based on knowledge of the reaction and the relevant product information documented in the SmPC which is stored in the site file and pharmacy file.

The event may be classed as either:

Expected: the AR is consistent with the toxicity of the IMP listed in the SmPC.

Unexpected: the AR is not consistent with the toxicity in the SmPC.

10.4.4 Assessment of Severity

The Investigator will make an assessment of severity for each AE/SAE and record this on the SAE form according to one of the following categories:

Mild: an event that is easily tolerated by the participant, causing minimal discomfort and not interfering with every day activities.

Moderate: an event that is sufficiently discomforting to interfere with normal everyday activities.

Severe: an event that prevents normal everyday activities.

Note: the term 'severe', used to describe the intensity, should not be confused with 'serious' which is a regulatory definition based on participant/event outcome or action criteria. For example, a headache may be severe but not serious, while a minor stroke is serious but may not be severe.

10.5 REPORTING OF SAES/SARS/SUSARS

Once the Investigator becomes aware that an SAE has occurred in a study participant, the information will be reported to the ACCORD Research Governance & QA Office **immediately or within 24 hours**. If the Investigator does not have all information regarding an SAE, they should not wait for this additional information before notifying ACCORD. The SAE report form can be updated when the additional information is received. Elective admission for LNG-IUS insertion or elective admission for hysterectomy would not need to be classified as a serious adverse event.

The SAE report will provide an assessment of causality and expectedness at the time of the initial report to ACCORD according to sections 10.4.2, Assessment of Causality and 10.4.3, Assessment of Expectedness.

The SAE form will be transmitted by fax to ACCORD on **+44 (0)131 242 9447** or submitted via email to Safety.Accord@ed.ac.uk. Only forms in a pdf format will be accepted by ACCORD via email.

Where missing information has not been sent to ACCORD after an initial report, ACCORD will contact the investigator and request the missing information.

All reports faxed to ACCORD and any follow up information will be retained by the Investigator in the Investigator Site File (ISF). Details received by ACCORD will be passed on to BCTU in their capacity as Coordinating Centre.

10.6 REGULATORY REPORTING REQUIREMENTS

The ACCORD Research Governance & QA Office is responsible for pharmacovigilance reporting on behalf of the co-sponsors (Edinburgh University and NHS Lothian).

The ACCORD Research Governance & QA Office has a legal responsibility to notify the regulatory competent authority and relevant ethics committee (Research Ethics Committee (REC) that approved the trial). Fatal or life threatening SUSARs will be reported no later than 7 calendar days and all other SUSARs will be reported no later than 15 calendar days after ACCORD is first aware of the reaction.

ACCORD will inform Investigators at participating sites of all SUSARs and any other arising safety information.

An Annual Safety Report/Development Safety Update Report will be submitted, by ACCORD, to the regulatory authorities and RECs listing all SARs and SUSARs.

10.7 FOLLOW UP PROCEDURES

After initially recording an unexpected AE or recording and reporting an SAE, the Investigator should make every effort to follow each event until a final outcome can be recorded or reported. The Investigator should follow each unexpected AE or SAE until the event has resolved, the event is assessed as stable by the Investigator, the participant is lost to follow up, or the participant withdraws consent. Follow up information on an SAE will be reported to the ACCORD office.

If, after follow up, resolution of an event cannot be established, an explanation should be recorded.

In the case of PAECs observed at 12 months in those allocated UPA, the women should be reassessed until an endometrial biopsy confirms the restoration of a normal endometrium.

10.8 PREGNANCY

Pregnancy will be considered an AE if women are compliant with either trial treatment. If a woman withdraws from trial treatment and conceives within 12 months of randomisation, this **will not** be considered an AE. However, the investigator will collect pregnancy information for any female participants while participating in the study. The Investigator will record the information on a Pregnancy Notification Form and submit this to the **Safety.Accord@ed.ac.uk** **or (0)131 242 9447** within 14 days of being made aware of the pregnancy. BCTU will follow-up any self-reported pregnancies with the women's GP or gynaecologist. All pregnant female participants will be followed

up until the outcome of the pregnancy is known. Details received by the UCON Trial Office will be passed on to ACCORD.

11 TRIAL MANAGEMENT AND OVERSIGHT ARRANGEMENTS

11.1 TRIAL MANAGEMENT GROUP

The trial will be coordinated by a Trial Management Group (TMG), consisting of the Chief Investigator, all other grant holders, the Trial Manager and Edinburgh based research nurse. The TMG will meet regularly, by teleconference or face to face.

11.2 THE UCON TRIAL OFFICE

The Trial Office at Birmingham Clinical Trials Unit (BCTU), University of Birmingham is responsible for the day to day management of the UCON Trial. The Trial Manager, based at BCTU, will oversee the study and will be accountable to the Chief Investigator. The Data Manager will be responsible for checking the data forms for completeness, plausibility and consistency. Any queries will be resolved by the Investigator or delegated member of the trial team.

The UCON Trial Office will also be responsible for providing all trial materials, including an Investigator Site File (ISF), with copies of all essential documents, and a trial stationary folders containing all required printed materials e.g. participant information sheets, consent forms. These will be supplied to each collaborating centre, after relevant local research governance approval has been obtained. Additional supplies of any printed material can be obtained on request. The Trial Office also provides the central randomisation service and is responsible for collection and checking of data (including reports of serious adverse events thought to be due to trial treatment), The Trial Office will help resolve any local problems that may be encountered in trial participation.

11.3 TRIAL STEERING COMMITTEE

A Trial Steering Committee (TSC) will be established to oversee the conduct and progress of the trial. Names and contact details are given on page iv.

The Trial Steering Committee (TSC) provides independent supervision for the trial, providing advice to the Chief and Co- Investigators and the Sponsor on all aspects of the trial and affording protection for patients by ensuring the trial is conducted according to the International Committee on Harmonisation Guidelines for Good Clinical Practice in Clinical Trials.

If the Chief and Co-Investigators are unable to resolve any concern satisfactorily, Principal Investigators, and all others associated with the study, may write through the Trial Office to the chairman of the TSC, drawing attention to any concerns they may have about the possibility of particular side-effects, or of particular categories of patient requiring special study, or about any other matters thought relevant.

The Trial office will forward TSC meeting minutes to the Sponsor and funder. Terms of reference of the Trial Steering Committee and the draft template for reporting will be agreed at the inaugural meeting.

11.4 DATA MONITORING COMMITTEE

An independent Data Monitoring Committee (DMC) will be established to oversee the safety of participants in the trial. Names and contact details are given on page iv.

If one treatment really is substantially better or worse than any other with respect to the primary outcome, then this may become apparent before the target recruitment has been reached. Alternatively, new evidence might emerge from other sources that any one treatment is definitely more, or less, effective than any other. To protect against this, during the main period of recruitment to the study, interim analyses of the primary outcome and adverse events will be supplied, in strict confidence, to the independent DMC, along with updates on results of other related studies, and any other analyses that the DMC may request. The DMC will advise the chair of the TSC if, in their view, any of the randomised comparisons in the trial have provided both (a) “proof beyond reasonable doubt”[1] that for all, or for some, types of patient one particular treatment is definitely indicated or definitely contraindicated in terms of a net difference in the major endpoints, and (b) evidence that might reasonably be expected to influence the patient management of many clinicians who are already aware of the other main trial results. The TSC can then decide whether to close or modify any part of the trial. Unless this happens, however, the TMG, TSC, the investigators and all of the central administrative staff (except the statisticians who supply the confidential analyses) will remain unaware of the interim results.

The BCTU Trial office will forward DMC open meeting minutes to the Sponsor and funder. Terms of reference of the Trial Steering Committee and the draft template for reporting will be agreed at the inaugural meeting.

11.5 INSPECTION OF RECORDS

Investigators and institutions involved in the study will permit trial related monitoring and audits on behalf of the sponsor, REC review, and regulatory inspection(s). In the event of an audit or monitoring, the Investigator agrees to allow the representatives of the sponsor direct access to all study records and source documentation. In the event of regulatory inspection, the Investigator agrees to allow inspectors direct access to all study records and source documentation.

11.6 RISK ASSESSMENT

An independent risk assessment will be performed by an ACCORD Clinical Trials Monitor to determine if monitoring is required and if so, at what level. An independent risk assessment will also be carried out by the ACCORD Quality Assurance Group to determine if an audit should be performed before, during or after the study and if so, at what locations and at what frequency.

1 Appropriate criteria of proof beyond reasonable doubt cannot be specified precisely, but a difference of at least $p<0.001$ (similar to a Haybittle-Peto stopping boundary) in an interim analysis of a major endpoint may be needed to justify halting, or modifying, the study prematurely. If this criterion were to be adopted, it would have the practical advantage that the exact number of interim analyses would be of little importance, so no fixed schedule is proposed.

11.7 STUDY MONITORING AND AUDIT

An ACCORD Clinical Trials Monitor or an appointed monitor may visit the Investigator site prior to the start of the study and/or during the course of the study if required, in accordance with the central monitoring plan.

Investigators and their host Trusts will be required to permit trial-related monitoring and audits to take place by either the ACCORD Clinical Trials Monitor and/or Trial Coordinator as and when required who would require direct access to source data and documents as requested. Risk assessment will determine if audit, by the ACCORD QA group, is required. Details will be captured in an audit plan. Audit of Investigator sites, study management activities and study collaborative units, facilities and 3rd parties may be performed.

The study will also adopt a centralised approach to monitoring data quality and compliance. A computer database will be constructed specifically for the trial data and will include range and logic checks to prevent erroneous data entry. Independent checking of data entry will be periodically undertaken on small sub-samples. The trial statistician will regularly check the balance of allocations by the stratification variables.

Trusts may also be subject to inspection by the Medicines and Healthcare Products Regulatory Agency and/ or by the Research and Development Manager of their own Trust and should do everything requested by the Chief Investigator in order to prepare and contribute to any inspection or audit. Trial participants will be made aware of the possibility of external audit of data they provide in the participant information sheet.

12 GOOD CLINICAL PRACTICE

12.1 ETHICAL CONDUCT

The study will be conducted in accordance with the principles of the International Conference on Harmonisation Tripartite Guideline for Good Clinical Practice (ICH GCP).

A favourable ethical opinion will be obtained from the appropriate REC and local R&D approval will be obtained prior to commencement of the study.

12.2 REGULATORY COMPLIANCE

The study will not commence until a Clinical Trial Authorisation (CTA) is obtained from the appropriate Regulatory Authority. The protocol and study conduct will comply with the Medicines for Human Use (Clinical Trials) Regulations 2004, as amended.

12.3 INVESTIGATOR RESPONSIBILITIES

The local Principal Investigator is responsible for the overall conduct of the study at the site and compliance with the protocol and any protocol amendments. In accordance with the principles of ICH GCP, the following areas listed in this section are also the responsibility of each Investigator. Responsibilities may be delegated to an appropriate member of study site staff. Delegated tasks must be documented on a Delegation Log and signed by all those named on the list prior to undertaking applicable study-related procedures.

12.3.1 Informed Consent

The Investigator is responsible for ensuring informed consent is obtained before any protocol specific procedures are carried out. The decision of a participant to participate in clinical research is voluntary and should be based on a clear understanding of what is involved.

Participants must receive adequate oral and written information – UCON Participant Information and Informed Consent Forms will be provided, with variations for those sites participating in the mechanistic sub-study. The oral explanation to the participant will be performed by the Investigator or qualified delegated person, and must cover all the elements specified in the Participant Information Sheet and Consent Form.

The participant must be given every opportunity to clarify any points they do not understand and, if necessary, ask for more information. The participant must be given sufficient time to consider the information provided. It should be emphasised that the participant may withdraw their consent to participate at any time without loss of benefits to which they otherwise would be entitled.

The participant will be informed and agree to their medical records being inspected by regulatory authorities and representatives of the sponsor(s) but understand that their name will not be disclosed outside the hospital.

The Investigator and the participant will sign and date the Informed Consent Form(s) to confirm that consent has been obtained. The participant will receive a copy of this document and with the original filed in the ISF and copies filed in the participant's medical notes and sent to the Trial Office.

12.3.2 Study Site Staff

The Investigator must be familiar with the IMP, protocol and the study requirements. It is the Investigator's responsibility to ensure that all staff assisting with the study are adequately informed about the trial treatments, protocol and their trial related duties.

Each participating centre should also designate at least one nurse as a UCON research nurse. This person would be responsible for ensuring that all eligible patients are considered for the study, that women are provided with study information sheets, and have an opportunity to discuss the study if required. The nurse will be responsible for ensuring the baseline participant questionnaire is completed and for randomisation.

A Delegation Log will be prepared for each site, detailing the responsibilities of each member of staff working on the trial.

12.3.3 Data Recording

The Principal Investigator is responsible for the quality of the data recorded in the clinician completed data forms at their site.

12.3.4 Investigator Documentation

The local Principal Investigator is responsible for maintenance of their site's Investigator Site File, including filing updates provided by the UCON Trial Office

Prior to beginning the study, each Investigator will be asked to provide particular essential documents to the BCTU, including but not limited to:

- An original signed Investigator's Declaration (as part of the Clinical Trial Agreement documents), detailing their commitment to accrual, compliance, GCP, confidentiality and publication. Deviations from the agreement will be monitored and the TSC will decide whether any action needs to be taken, e.g. withdrawal of funding, suspension of centre.
- Curriculum vitae (CV) signed and dated by the Investigator indicating that it is accurate and current.

The UCON Trial Office will ensure all other documents required by ICH GCP are retained in a Trial Master File (TMF), where required, and that appropriate documentation is available in local ISFs.

12.3.5 GCP Training

All study staff must hold evidence of appropriate GCP training.

12.3.6 Confidentiality

All endometrial biopsy samples, data collection forms and patient questionnaires must be identified in a manner designed to maintain participant confidentiality. All records must be kept in a secure storage area with limited access. Clinical information will not be released without the written permission of the participant. All investigators and study site staff involved with this study may not disclose or use for any purpose other than performance of the study, any data, record, or other unpublished, confidential information disclosed to those individuals for the purpose of the study. Prior written agreement from the sponsor or its designee must be obtained for the disclosure of any said confidential information to other parties.

All personal information received in paper format for the trial will be held securely and treated as strictly confidential according to BCTU policies. All staff involved in the UCON Trial (clinical, academic, BCTU) share the same duty of care to prevent unauthorised disclosure of personal information. No data that could be used to identify an individual will be published. Data will be stored on a secure server at Birmingham Clinical Trials Unit (BCTU) under the provisions of the Data Protection Act and/or applicable laws and regulations.

12.3.7 Data Protection

All Investigators and study site staff involved with this study must comply with the requirements of the Data Protection Act 1998 with regard to the collection, storage, processing and disclosure of personal information and will uphold the Act's core principles. Access to collated participant data will be restricted to designated staff at the UCON Trial Office, clinicians treating the participants, representatives of the sponsor(s) and representatives of regulatory authorities.

Computers used to collate the data will have limited access measures via user names and passwords.

Published results will not contain any personal data that could allow identification of individual participants.

13 STUDY CONDUCT RESPONSIBILITIES

13.1 PROTOCOL AMENDMENTS

Any changes in research activity, except those necessary to remove an apparent, immediate hazard to the participant in the case of an urgent safety measure, must be reviewed and approved by the Chief Investigator.

Amendments to the protocol must be submitted in writing to the appropriate REC, Regulatory Authority and local R&D for approval prior to participants being enrolled into an amended protocol.

13.2 PROTOCOL VIOLATIONS AND DEVIATIONS

Protocol deviations will be recorded in a protocol deviation log and logs will be submitted to the sponsors every 3 months. Each protocol violation will be reported to the sponsor within 3 days of becoming aware of the violation. Deviation logs / violation forms will be transmitted via email to QA@accord.scot. Only forms in a pdf format will be accepted by ACCORD via email. Forms may also be sent by fax to ACCORD on +44 (0)131 242 9447 or may be submitted by hand to the office. Where missing information has not been sent to ACCORD after an initial report, ACCORD will contact the Investigator and request the missing information. The Investigator must respond to these requests in a timely manner.

13.3 SERIOUS BREACH REQUIREMENTS

A serious breach is a breach which is likely to effect to a significant degree:

1. the safety or physical or mental integrity of the participants of the trial; or
2. the scientific value of the trial.

If a potential serious breach is identified by the Chief investigator, Principal Investigator or delegates, the co-sponsors (QA@accord.scot) must be notified within 24 hours. It is the responsibility of the co-sponsors to assess the impact of the breach on the scientific value of the trial, to determine whether the incident constitutes a serious breach and report to regulatory authorities and research ethics committees as necessary. Details received by ACCORD will be passed on to BCTU in their capacity as Coordinating Centre.

13.4 STUDY RECORD RETENTION

In line with the Medicines for Human Use (Clinical Trials) Regulations, once data collection is complete on all participants, at the end of the study, all data will be stored for at least 15 years. This will allow adequate time for review and reappraisal, and form the basis for further follow-up research. Any queries or concerns about the data, conduct or conclusions of the trial can also be resolved in this time. When the minimum retention period has elapsed, study documentation will not be destroyed without permission from the sponsor.

13.5 END OF STUDY

The end of study is defined as the completion of the last participant's 12-month follow-up assessment unless PAECs are detected at this time. Should PAECs be diagnosed at 12 months then the end of study is determined by an additional biopsy(ies) and the resolution of the PAECs at either 15 or 18 months. The funder and/or Investigators and/or the trial steering committee and/or the co-sponsor(s) have the right at any time to terminate the study for clinical or administrative reasons.

The end of the study will be reported to the REC, Regulatory Authority, R&D Office(s) and co-sponsors within 90 days, or 15 days if the study is terminated prematurely. The Investigators will inform participants of the premature study closure and ensure that the appropriate follow up is arranged for all participants involved. End of study notification will be reported to the co-sponsors via email to researchgovernance@ed.ac.uk.

In accordance with ACCORD SOP CR011, a Clinical Study Report (CSR) will be provided to the Sponsor (QA@accord.scot) and REC within 1 year of the end of the study.

Upon completion of the study, the Investigator will upload clinical trial results onto the EudraCT database on behalf of the Sponsor.

13.6 CONTINUATION OF DRUG FOLLOWING THE END OF STUDY

Although UPA is a licensed drug for the treatment of uterine fibroids, it is not routinely prescribed for the treatment of heavy menstrual bleeding. Justification for the use of UPA after the trial will have to be discussed at the centre on a case-by-case basis; this discussion will be outside the remit of the trial.

13.7 INSURANCE AND INDEMNITY

The co-sponsors are responsible for ensuring proper provision has been made for insurance or indemnity to cover their liability and the liability of the Chief Investigator and staff.

The following arrangements are in place to fulfil the co-sponsors' responsibilities:

- The Protocol has been designed by the Chief Investigator and researchers employed by the University and collaborators. The University has insurance in place (which includes no-fault compensation) for negligent harm caused by poor protocol design by the Chief Investigator and researchers employed by the University.

- Sites participating in the study will be liable for clinical negligence and other negligent harm to individuals taking part in the study and covered by the duty of care owed to them by the sites concerned. The co-sponsors require individual sites participating in the study to arrange for their own insurance or indemnity in respect of these liabilities.

- Sites which are part of the United Kingdom's Nation Health Service will have the benefit of NHS Indemnity.

- The manufacturer supplying IMP has accepted limited liability related to the manufacturing and original packaging of the study drug and to the losses, damages, claims or liabilities incurred by study participants based on known or unknown Adverse Events which arise out

of the manufacturing and original packaging of the study drug, but not where there is any modification to the study drug (including without limitation re-packaging and blinding).

14 REPORTING, PUBLICATIONS AND NOTIFICATION OF RESULTS

14.1 AUTHORSHIP POLICY

Ownership of the data arising from this study resides with the grant holders. On completion of the study, the study data will be analysed and tabulated, and a clinical study report will be prepared in accordance with ICH guidelines. A writing committee may be established to prepare the report.

The main report of the trial will be published in the name of the UCON Collaborative Group, acknowledging the writing group as authors. Subsequent publications should also be published in the UCON Collaborative Group name, but those academics who contribute to specific aspects may be listed as authors.

14.2 PUBLICATION

The clinical study report will be used for publication and presentation at scientific meetings. Investigators have the right to publish orally or in writing the results of the study.

A meeting will be held after the end of the study to allow discussion of the main results among the collaborators prior to publication. The success of the study depends entirely on the wholehearted collaboration of a large number of doctors, nurses and others. For this reason, chief credit for the main results will be given not to the committees or central organisers but to all those who have collaborated in the study, who will be listed as members the UCON Collaborative Group in all publications. Centres will be permitted to publish data obtained from participants in the UCON Trial that use trial outcome measures but do not relate to the trial randomised evaluation and hypothesis.

15 REFERENCES

1. Rahn D, Abed H, Sung V, Matteson K, Rogers R, Morrill M, et al. Systematic review highlights difficulty interpreting diverse clinical outcomes in abnormal uterine bleeding trials. J Clin Epidemiol. 2011;64(3):293-300.
2. Higham J, O'Brien P, Shaw R. Assessment of menstrual blood loss using a pictorial. chart Br J Obstet Gynaecol. 1990;97.((8)):734-9.
3. Spies J, Coyne K, Guaou Guaou N, Boyle D, Skyrnarz-Murphy K, Gonzalves S. The UFSQOL, a new disease-specific symptom and health-related quality of life questionnaire for leiomyomata. Obstet Gynecol. 2002;99((2)):290-300.
4. Thirlaway K, Fallowfield L, Cuzick J. The Sexual Activity Questionnaire: a measure of women's sexual functioning. Qual Life Res. 1996;5 ((1)):81-90.
5. Group E. EuroQol--a new facility for the measurement of health-related quality of life. Health Policy. 1990;16(3):199-208.
6. Al-Janabi H, Flynn TN, Coast J. Development of a self-report measure of capability wellbeing for adults: the ICECAP-A. Qual Life Res. 2012;21(1):167-76.
7. Rahn DD, Abed H, Sung VW, Matteson KA, Rogers RG, Morrill MY, et al. Systematic review highlights difficulty interpreting diverse clinical outcomes in abnormal uterine bleeding trials. J Clin Epidemiol. 2011;64(3):293-300.
8. Abdel-Aleem H, d'Arcangues C, Vogelsong KM, Gulmezoglu AM. Treatment of vaginal bleeding irregularities induced by progestin only contraceptives. Cochrane Database Syst Rev. 2007(4):CD003449.
9. Gupta J, Kai J, Middleton L, Pattison H, Gray R, Daniels J, et al. Levonorgestrel intrauterine system versus medical therapy for menorrhagia. N Engl J Med. 2013;368(2):128-37.
10. Donnez J, Tomaszewski J, Vazquez F, Bouchard P, Lemieszczuk B, Baro F, et al. Ulipristal acetate versus leuprolide acetate for uterine fibroids. N Engl J Med. 2012;366(5):421-32.
11. Mutter GL, Bergeron C, Deligdisch L, Ferenczy A, Glant M, Merino M, et al. The spectrum of endometrial pathology induced by progesterone receptor modulators. Mod Pathol. 2008;21(5):591-8.
12. Williams AR, Bergeron C, Barlow DH, Ferenczy A. Endometrial morphology after treatment of uterine fibroids with the selective progesterone receptor modulator, ulipristal acetate. Int J Gynecol Pathol. 2012;31(6):556-69.
13. Pohl O, Williams AR, Bergeron C, Gotteland JP. A 39-week oral toxicity study of ulipristal acetate in cynomolgus monkeys. Regul Toxicol Pharmacol. 2013;66(1):6-12.
14. Mutter GL, Ince TA, Baak JP, Kust GA, Zhou XP, Eng C. Molecular identification of latent precancers in histologically normal endometrium. Cancer Res. 2001;61(11):4311-4.
15. Daikoku T, Hirota Y, Tranguch S, Joshi AR, DeMayo FJ, Lydon JP, et al. Conditional loss of uterine Pten unfailingly and rapidly induces endometrial cancer in mice. Cancer Res. 2008;68(14):5619-27.
16. Mutter GL, Lin MC, Fitzgerald JT, Kum JB, Baak JP, Lees JA, et al. Altered PTEN expression as a diagnostic marker for the earliest endometrial precancers. J Natl Cancer Inst. 2000;92(11):924-30.
17. Orbo A, Rise CE, Mutter GL. Regression of latent endometrial precancers by progestin infiltrated intrauterine device. Cancer Res. 2006;66(11):5613-7.
18. Zheng W, Baker HE, Mutter GL. Involution of PTEN-null endometrial glands with progestin therapy. Gynecol Oncol. 2004;92(3):1008-13.

19. Shaw RW, Brickley MR, Evans L, Edwards MJ. Perceptions of women on the impact of menorrhagia on their health using multi-attribute utility assessment. Br J Obstet Gynaecol. 1998;105(11):1155-9.
20. Higham JM, O'Brien PM, Shaw RW. Assessment of menstrual blood loss using a pictorial chart. Br J Obstet Gynaecol. 1990;97(8):734-9.
21. Spies JB, Coyne K, Guaou Guaou N, Boyle D, Skyrnarz-Murphy K, Gonzalves SM. The UFS-QOL, a new disease-specific symptom and health-related quality of life questionnaire for leiomyomata. Obstet Gynecol. 2002;99(2):290-300.
22. Thirlaway K, Fallowfield L, Cuzick J. The Sexual Activity Questionnaire: a measure of women's sexual functioning. Qual Life Res. 1996;5(1):81-90.
23. Herdman M, Gudex C, Lloyd A, Janssen M, Kind P, Parkin D, et al. Development and preliminary testing of the new five-level version of EQ-5D (EQ-5D-5L). Qual Life Res. 2011;20(10):1727-36.
24. Al-Janabi H, Coast J. ICECAP-A measure V2. Social Science and Medicine. 2008.

16 Appendix 1: TRIAL SCHEMA (Prior to USM)

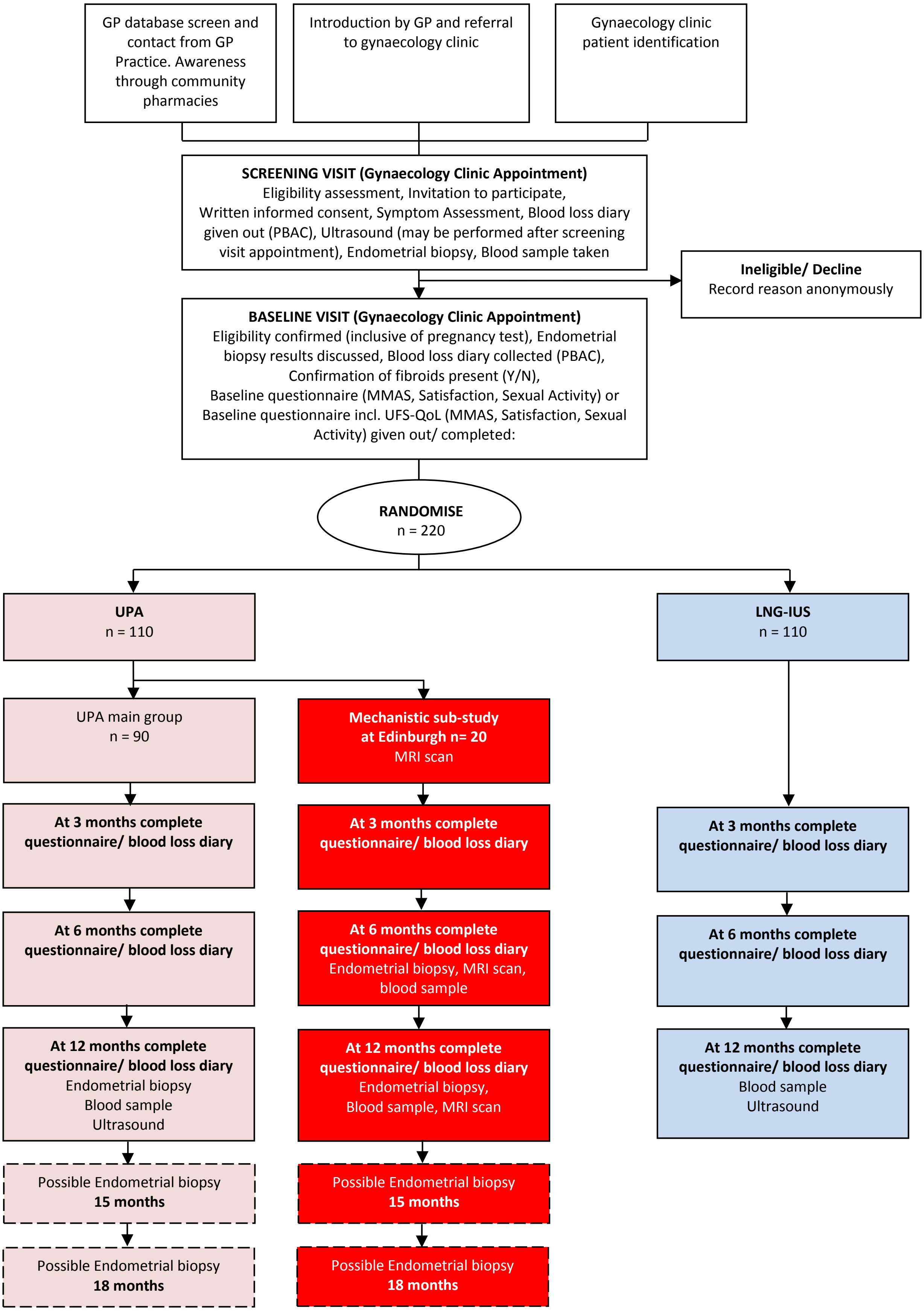

Supplement: UCON Study protocol [file mmc2.pdf]
